# Adenylyl cyclase isoform 1 contributes to sinoatrial node automaticity via functional microdomains

**DOI:** 10.1172/jci.insight.162602

**Published:** 2022-11-22

**Authors:** Lu Ren, Phung N. Thai, Raghavender Reddy Gopireddy, Valeriy Timofeyev, Hannah A. Ledford, Ryan L. Woltz, Seojin Park, Jose L. Puglisi, Claudia M. Moreno, Luis Fernando Santana, Alana C. Conti, Michael I. Kotlikoff, Yang Kevin Xiang, Vladimir Yarov-Yarovoy, Manuela Zaccolo, Xiao-Dong Zhang, Ebenezer N. Yamoah, Manuel F. Navedo, Nipavan Chiamvimonvat

**Affiliations:** 1 Department of Internal Medicine, Division of Cardiovascular Medicine, UCD, Davis, California, USA.; 2 Stanford Cardiovascular Institute, Stanford University School of Medicine, Stanford, California, USA.; 3 Department of Veteran Affairs, Northern California Health Care System, Sacramento, California, USA.; 4 Department of Pharmacology, UCD, Davis, California, USA.; 5 Department of Physiology and Cell Biology, University of Nevada, Reno, Reno, Nevada, USA.; 6 Prestige Biopharma Korea, Myongjigukje 7-ro, Gangseo-gu, Busan, South Korea.; 7 College of Medicine. California North State University, Sacramento, California, USA.; 8 Department of Physiology and Membrane Biology, UCD, Davis, California, USA.; 9 Department of Physiology and Biophysics, University of Washington School of Medicine, Seattle, Washington, USA.; 10 Research & Development Service, John D. Dingell VA Medical Center, and; 11 Department of Psychiatry and Behavioral Neurosciences, Wayne State University School of Medicine, Detroit, Michigan, USA.; 12 College of Veterinary Medicine, Cornell University, Ithaca, New York, USA.; 13 Department of Physiology, Anatomy and Genetics, University of Oxford, United Kingdom.

**Keywords:** Cardiology, Arrhythmias, Calcium signaling, Cardiovascular disease

## Abstract

Sinoatrial node (SAN) cells are the heart’s primary pacemaker. Their activity is tightly regulated by β-adrenergic receptor (β-AR) signaling. Adenylyl cyclase (AC) is a key enzyme in the β-AR pathway that catalyzes the production of cAMP. There are current gaps in our knowledge regarding the dominant AC isoforms and the specific roles of Ca^2+^-activated ACs in the SAN. The current study tests the hypothesis that distinct AC isoforms are preferentially expressed in the SAN and compartmentalize within microdomains to orchestrate heart rate regulation during β-AR signaling. In contrast to atrial and ventricular myocytes, SAN cells express a diverse repertoire of ACs, with AC_I_ as the predominant Ca^2+^-activated isoform. Although *AC_I_-*KO (*AC_I_^–/–^*) mice exhibit normal cardiac systolic or diastolic function, they experience SAN dysfunction. Similarly, SAN-specific CRISPR/Cas9-mediated gene silencing of *AC_I_* results in sinus node dysfunction. Mechanistically, hyperpolarization-activated cyclic nucleotide-gated 4 (HCN4) channels form functional microdomains almost exclusively with AC_I_, while ryanodine receptor and L-type Ca^2+^ channels likely compartmentalize with AC_I_ and other AC isoforms. In contrast, there were no significant differences in T-type Ca^2+^ and Na^+^ currents at baseline or after β-AR stimulation between WT and ACI^–/–^ SAN cells. Due to its central characteristic feature as a Ca^2+^-activated isoform, AC_I_ plays a unique role in sustaining the rise of local cAMP and heart rates during β-AR stimulation. The findings provide insights into the critical roles of the Ca^2+^-activated isoform of AC in sustaining SAN automaticity that is distinct from contractile cardiomyocytes.

## Graphical abstract

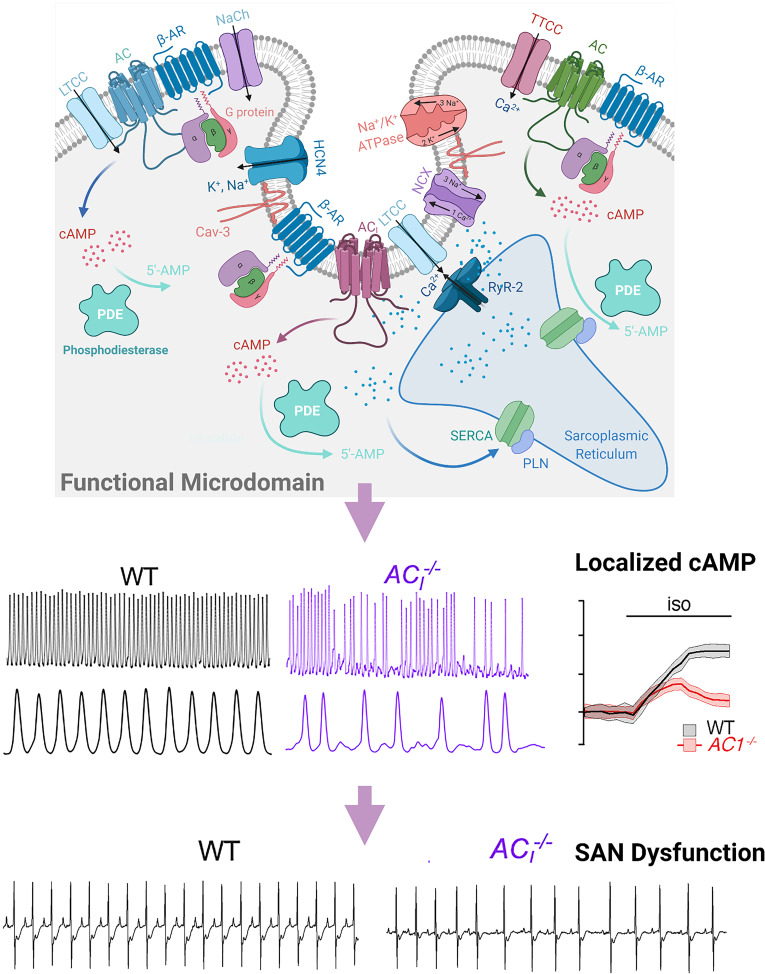

## Introduction

Sinoatrial node (SAN) dysfunction (SND) results in an inability of the heart’s natural pacemaker cells to produce a normal rhythm. The prevalence is approximately 1 per 1,000 person years (1, 2) with a projected incidence of 172,000 people per year by 2060 (3), due to an ageing population. SND has a broad etiologic spectrum and affects children with congenital heart diseases, as well as the elderly (4, 5). However, there are significant knowledge gaps in our current understanding of SAN functions and its regulation.

SAN, the pacemaker of the heart, is the primary determinant of heart rate (HR) (6, 7) and is tightly regulated by β-adrenergic receptor (β-AR) signaling (8–10). Adenylyl cyclase (AC) is a key enzyme in the β-AR pathway that catalyzes the reaction to convert ATP into cAMP, resulting in a cascade of downstream effects (9, 11, 12). ACs are genetically diverse and encoded by 10 distinct genes. Nine ACs are transmembrane proteins, while 1 is a cytosolic form (11, 13).

The function of different isoforms of AC is directly dependent on the concentrations of intracellular Ca^2+^; AC_I_ (*K*_d_ = 100 nM) (14) and AC_VIII_ (*K*_d_ = 500 nM) (15) are Ca^2+^-activated isoforms, while physiological concentrations of Ca^2+^ inhibit AC_V-VI_ (16). The genetic diversity suggests that different isoforms may serve distinct functions in specific regions of the body. The distribution of the AC isoforms may differ significantly between the SAN and working cardiomyocytes. In ventricular cardiomyocytes, the 2 Ca^2+^-inhibited isoforms, AC_V_ and AC_VI_, are the 2 main isoforms (17). In contrast to ventricular myocytes, previous studies suggest the expression of Ca^2+^-activated AC isoforms in the SAN (18, 19). However, the functional roles and compartmentalization of Ca^2+^-activated AC isoforms in the SAN remain incompletely understood.

Ca^2+^ is an essential key modulator of the SAN’s pacemaker potential via the Ca^2+^ clock (20), during which Ca^2+^ is spontaneously released from the sarcoplasmic reticulum (SR) via ryanodine receptor 2 (RyR2), triggering the extrusion of Ca^2+^ from the cytosol via the Na^+^-Ca^2+^ exchanger (NCX). NCX exchanges 1 Ca^2+^ ion for 3 Na^+^ ions, generating an overall net inward current that contributes to diastolic depolarization (20).

To gain a mechanistic understanding of how the SAN function is regulated, we tested the hypothesis that distinct AC isoforms are preferentially expressed and compartmentalized in the SAN to serve a specialized function. Specifically, activated AC_I_ increases cAMP concentrations, leading to an elevation of intracellular Ca^2+^ (Ca^2+^_i_) through Ca^2+^ channels. The subsequent increase in Ca^2+^_i_ activates AC_I_ in a positive-feedback loop, while the rise in Ca^2+^_i_ provides negative feedback to other AC isoforms such as AC_V–VI_. Our study aims to elucidate the critical role of the Ca^2+^-activated AC_I_ isoform in the SAN and provides insights into the development of targeted therapeutics, specific to pacemaker cells, without interfering with contractile myocytes.

## Results

### AC_I_ is the predominant Ca^2+^-activated isoform in the SAN, forming microdomains with key Ca^2+^ handling proteins.

**Figure 1:**
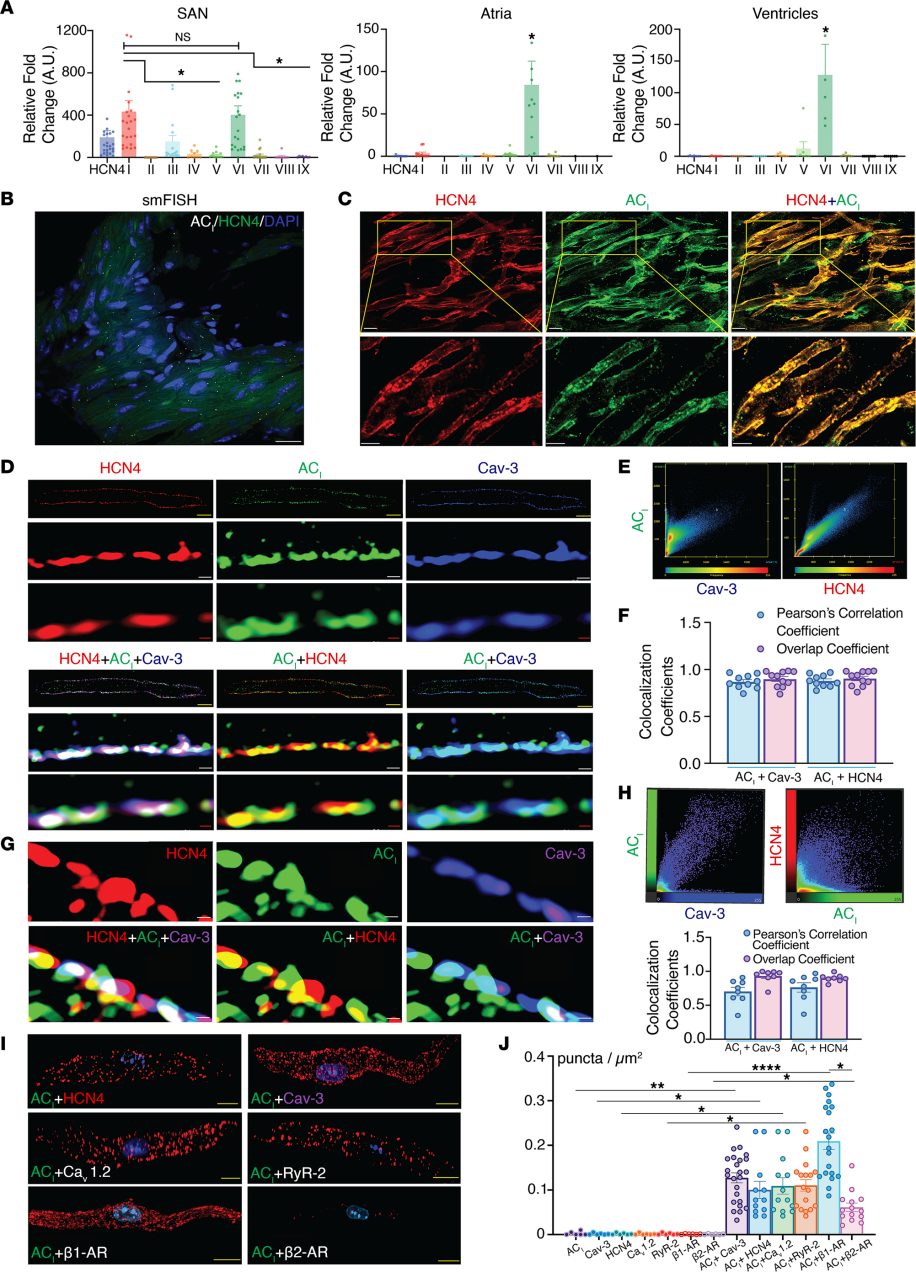
AC_I_ is the predominant Ca^2+^-activated isoform in the SAN, forming microdomains with key Ca^2+^ handling proteins and HCN4 channels. Summary data of relative abundance of AC_I-VIII_ in single cells isolated from 3 regions of the heart. (**A**) Single-cell qPCR from SAN, atria, and ventricles (*n* = 23, 11, 7 cells, respectively, from *n* = 3–5 mice for each group). (**B**) Representative smFISH images of AC_I_ mRNA expressions in the SAN tissue. HCN4 (green) was used as a counterstain for SAN tissue, and DAPI (blue) was used to stain the nuclei. Images were obtained from SAN tissues that were cryo-sectioned (10 μm) onto super-frost slides. Approximately 20–30 sections were obtained for each sample. Scale bar: 20 μm. (**C**) Representative high-resolution Airyscan immunofluorescence images of whole-mount SAN tissues, stained with anti-HCN4 (red) and AC_I_ (green) antibodies. Images at higher magnifications are shown in the second rows for each group. Scale bars: 5 μm. (**D**) Representative high-resolution Airyscan images of isolated SAN cells, stained with anti-HCN4 (red), AC_I_ (green), and Cav-3 (blue) antibodies. Images at higher magnifications are shown in the second and third rows for each group. Yellow, white, and red scale bars: 10, 0.4, and 0.2 μm, respectively. (**E**) Scatterplot analyses for the colocalization of AC_I_ with Cav-3 and AC_I_ with HCN4 from Airyscan confocal microscopic images, where the fluorescence intensity values of the 2 fluorochromes for each pixel are plotted against each other. (**F**) Additional analyses for the colocalization of AC_I_ and Cav-3 and of AC_I_ and HCN4 were performed using Pearson’s correlation coefficients (the ratio between the covariance of 2 variables and the product of their SDs) and overlap coefficients (the proportion of overlap between 2 probability distributions, as a measure of the similarity between distributions) from Airyscan images (*n* = 10–11 cells from 4 mice). (**G**) Representative super-resolution STED images of isolated SAN cells, triple-labeled for HCN4 (red), AC_I_ (green), and Cav-3 (purple). Scale bar: 0.2 μm. (**H**) Upper panel: Scatterplot analyses for the colocalization of AC_I_ with Cav-3 and AC_I_ with HCN4 from STED images. Lower panel: Additional analyses for the colocalization of AC_I_ and Cav-3 and of AC_I_ and HCN4 were performed using Pearson’s correlation and overlap coefficients from STED images (*n* = 8 cells from 3 mice). (**I**) Representative 3D rendering of PLA in SAN cells for AC_I_ with HCN4, Cav-3, Ca_v_1.2, RyR2, β_1_-AR, and β_2_-AR. Supplemental Video 1 shows a 3D rendering of AC_I_ and Cav-3. Scale bar: 5 μm. (**J**) Summary of PLA data, *n* = 12–24 cells from 4–6 mice per group. Data are expressed as mean ± SEM. *P < 0.05, **P < 0.01, and ****P < 0.0001 by 1-way ANOVA for multiple comparisons, followed by Kruskal-Wallis post hoc analyses.

To identify different AC isoforms expressed in SAN cells compared with atrial and ventricular myocytes, we performed single-cell quantitative PCR (qPCR). AC_I_ and AC_VI_ were the most abundant isoforms in the SAN, followed by AC_III_, AC_IV_, and AC_VII_ (Figure 1A and Supplemental Figure 1A; supplemental material available online with this article; https://doi.org/10.1172/jci.insight.162602DS1). In contrast, AC_VI_ was the most abundant isoform in both atria and ventricles. We used the HCN4 channel as a marker to accurately identify SAN cells. Single-molecule FISH (smFISH) corroborated single-cell qPCR data (Figure 1B). We detected both AC_I_ and AC_VI_ mRNA expression in the SAN tissue. HCN4 was used for counterstain, and the GAPDH probe was used as a positive control (Supplemental Figure 1, B–D). Whole-mount SAN tissues were used to document the protein expression of AC_I_ and HCN4 in the SAN using immunofluorescence high-resolution microscopy (Figure 1C). Specificity of anti-AC_I_ antibody was documented using whole-mount SAN tissues isolated from WT and *AC_I_^–/–^* mice (21, 22) (Supplemental Figure 1E).

Caveolin-3 (Cav-3) is a critical scaffolding protein, involved in the organization of signaling microdomains (23). We have previously shown that the AC_V_ isoform colocalizes and interacts with Cav-3 to regulate Ca^2+^ current in ventricular myocytes (17). We performed immunofluorescence imaging of SAN cells, triple labeled for AC_I_, Cav-3, and HCN4, using confocal microscopy coupled with an Airyscan module, with a resolution of ~120 nm (Figure 1D). AC_I_ colocalizes with Cav-3 and HCN4, as evidenced by the high Pearson’s correlation coefficient and overlap coefficient (Figure 1, E and F). Additionally, stimulated emission depletion (STED) microscopy with a resolution of approximately 50 nm was used to document the colocalization at a higher spatial resolution. In agreement with Airyscan images, STED microscopy images of the SAN cells and subsequent Pearson’s correlation coefficient and overlap coefficient analysis revealed that these proteins form clusters with a high degree of overlap at the plasma membrane (Figure 1, G and H). Specificity of anti-AC_I_ antibody was demonstrated using SAN cells isolated from *AC_I_*-KO mice (21, 22) (Supplemental Figure 2, A and B).

Proximity ligation assay (PLA) was further used as a complementary technique to determine if the 2 proteins of interest are ≤ 40 nm apart (Figure 1I). Red puncta represent close association of AC_I_ with specific proteins, and nuclei were stained with DAPI. The numbers of puncta per unit area were significantly increased when AC_I_ was costained with HCN4, Cav-3, voltage-gated Ca^2+^ channel (Ca_v_1.2), RyR2, β_1_-AR, and β_2_-AR compared with negative controls (where only 1 antibody was used) (Figure 1J and Supplemental Figure 2, C–E; *P* < 0.001 compared with negative controls). Moreover, there was a significantly higher number of punta per unit area between AC_I_ and β_1_-AR compared with AC_I_ and β_2_-AR (Figure 1J). These results suggest that the Ca^2+^-activated AC_I_ isoform is predominantly expressed in the SAN and localizes within microdomains with key Ca^2+^ handling proteins, HCN4 channels, and β_1_-AR.

### AC_I_^–/–^ mice exhibit SND.

**Figure 2:**
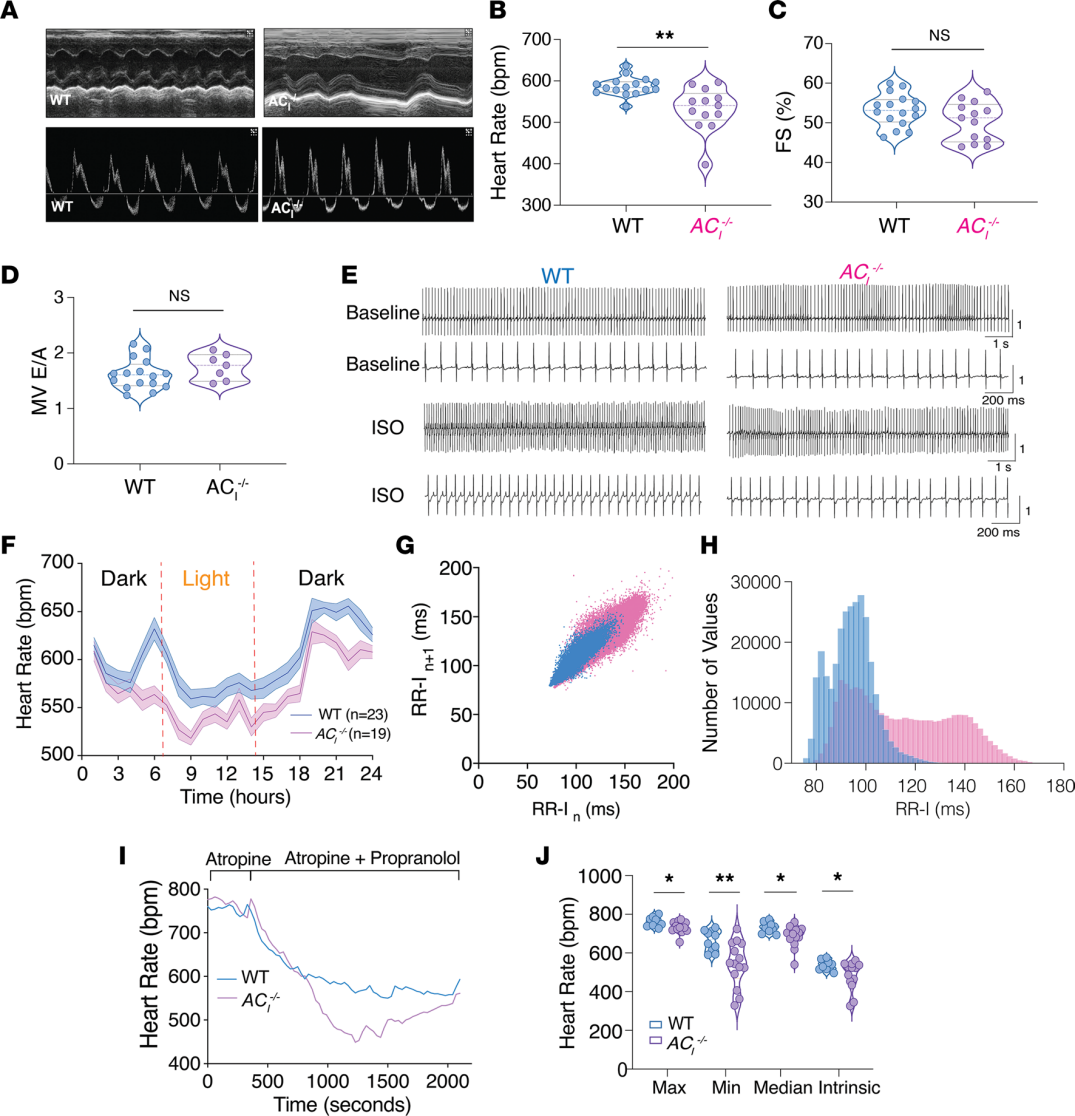
*AC_I_*^–/–^ mice exhibit sinus node dysfunction. (**A**–**D**) Representative M-mode echocardiographic images (**A**) for WT and *AC_I_^–/–^* mice. Summary data from echocardiography for heart rate in beats per minute (bpm) (**B**), fractional shortening (FS) (**C**), and mitral valve (MV) E/A ratio (**D**). (**E**) Representative ECG tracings of WT and *AC_I_^–/–^* mice at baseline and after β-AR stimulation. (**F**) Heart rates (bpm) over 24-hour period are plotted for WT and *AC_I_^–/–^* mice (data are expressed as mean ± SEM; *n* = 23 and 19 mice for WT and *AC_I_^–/–^*, respectively). Heart rates were averaged after every hour for a 24-hour recording. (**G**) Heart rate variability, plotted as RR intervals (RR-I) for *n* beat and *n* + 1 beat in ms. (**H**) Heart rate histograms with numbers of values for each RR-I. (**I**) Representative heart rate traces of WT and *AC_I_^–/–^* mice after injection of atropine, followed by propranolol to block autonomic nervous system. (**J**) Summary data of the maximum, minimum, median, and intrinsic heart rates from **I** (*n* = 10 and 14 mice for WT and *AC_I_^–/–^*, respectively). Summary data in **B**–**D** and **J** are shown as violin plots with symbols within the plots representing individual data points with median as well as quartiles indicated as dashed line. *P < 0.05 and **P < 0.01 by Student’s *t* test.

How the Ca^2+^-activated AC_I_ isoform contributes to SAN automaticity, in addition to the known AC isoforms, remains unclear (24). We used *AC_I_^–/–^* mice (21, 22) and used echocardiography to quantify cardiac dimensions and function in *AC_I_^–/–^* mice compared with WT animals (Supplemental Figure 3). Genotyping was performed in all mice (Supplemental Figure 4). To assess systolic function, M-mode images were acquired at the parasternal short axis in conscious WT and *AC_I_^–/–^* mice (Figure 2A). Color and pulse wave Doppler were used to evaluate diastolic function in anesthetized mice, by assessing the ratio of blood flow velocity through the mitral valve (MV) during early diastolic filling (E wave) and late diastolic filling (A wave) (Figure 2A). *AC_I_^–/–^* mice showed evidence of sinus bradycardia and sinus arrhythmias (Figure 2B); HRs were 532 ± 15 compared with 587 ± 6 bpm for *AC_I_^–/–^* and WT mice, respectively (*n* = 13 and 16, **P < 0.01). However, there were no significant differences in systolic or diastolic function between the 2 groups (Figure 2, C and D, and Supplemental Figure 3). Normalized heart weight/body weight ratio was not different between WT and *AC_I_^–/–^* mice, with no evidence of cardiac fibrosis (Supplemental Figure 3). There was no compensatory upregulation of AC_V_ and AC_VI_ mRNA expression in *AC_I_^–/–^* mice compared with WT controls (Supplemental Figure 1A).

### AC_I_^–/–^ mice exhibit blunted HR responses to β-AR stimulation.

Ambulatory ECG recordings were performed to assess baseline HR, followed by β-AR stimulation with isoproterenol (ISO) or autonomic nervous system (ANS) blockade with atropine and propranolol (Figure 2, E and F). There were diurnal variations with lower HRs during the light than the dark cycles. In addition, HRs in *AC_I_^–/–^* mice were significantly lower than in WT animals, and the differences were most pronounced during the hours when the mice were active. A representative daytime (7 a.m.–7 p.m.) HR variability scatter plot shows that there were more variations in HRs (Figure 2G) with significantly higher RR intervals (RR-I, lower HRs) in the histogram plots from *AC_I_^–/–^* compared with those from WT mice (Figure 2H). Further analyses revealed that this abnormality persisted at night (7 p.m.–7 a.m.), when the mice exhibited the highest activity level (Supplemental Figure 5).

To determine the intrinsic HR, we administered 2 mg/kg atropine followed by 1 mg/kg propranolol i.p. (Figure 2I). The HR after β-AR stimulation (maximum, minimum, and median HR) and after ANS blockade (intrinsic HR; 536 ± 8 bpm for WT versus 482 ± 21 bpm for *AC_I_^–/–^*; *P* = 0.0426) were significantly impaired in *AC_I_^–/–^* relative to WT mice (Figure 2J).

### SAN-specific CRISPR/Cas9-mediated AC_I_ gene silencing causes SND.

As a complementary experiment to the global KO model, we took advantage of CRISPR/Cas9 gene silencing techniques (25, 26) to generate a SAN-specific KO of the *AC_I_* gene in a transgenic HCN4-GCaMP8 background (Supplemental Figures 4, 6, and 7). Control constructs contained scrambled sequences and both constructs contained a reporter gene (mCherry). The constructs in the liposome were delivered directly onto the SAN region (27, 28).

**Figure 3:**
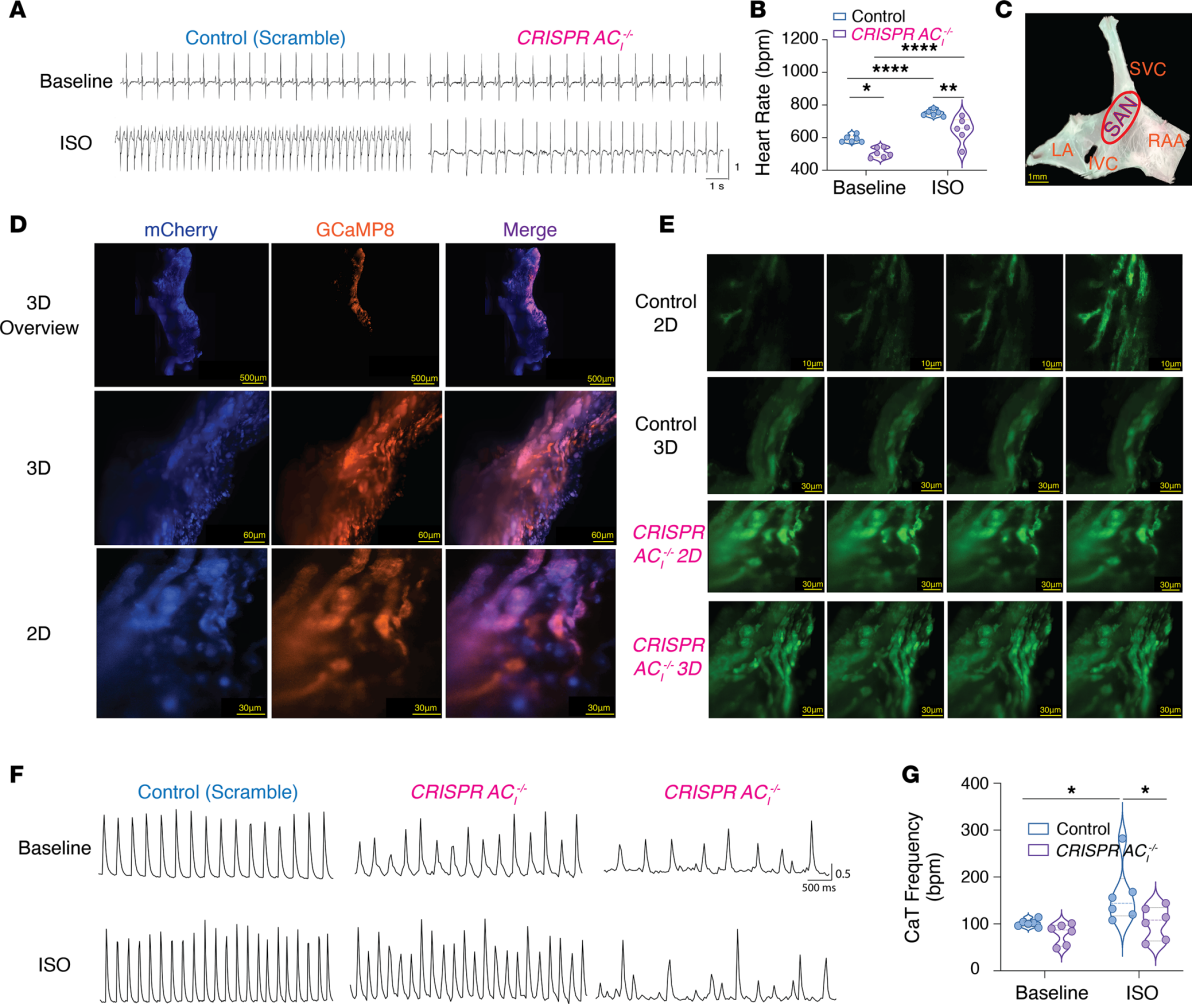
SAN-specific CRISPR/Cas9-mediated *AC_I_* gene silencing (*CRISPR AC_I_^–/–^*) exhibit sinus node dysfunction. (**A**) Representative surface ECG recordings for SAN-specific CRISPR/Cas9-mediated *AC_I_* gene silencing mice at baseline and after ISO stimulation. Delivery of scrambled sgRNA was used as control. (**B**) Summary data for heart rate (bpm) at baseline and after ISO injection (*n* = 6 mice for each group). (**C**) SAN isolated from liposome-treated mice with the following landmarks. LA, left atrium; RAA, right atrial appendage; IVC, inferior vena cava; SVC, superior vena cava. (**D**) Representative images of SAN tissue with mCherry signals from the reporter gene and green fluorescence signals from the genetically encoded HCN4-GCaMP8 transgenic mice. (**E**) Ca^2+^ signals are shown for WT and SAN-specific CRISPR/Cas9-mediated *AC_I_* gene silencing SAN tissues. Supplemental Video 2 shows Ca^2+^ signals from a SAN tissue from SAN-specific CRISPR/Cas9-mediated *AC_I_* gene silencing. (**F**) Representative CaT traces from SAN-specific CRISPR/Cas9-mediated *AC_I_* gene silencing SAN tissue compared with control mice (treated with scrambled sgRNA) at baseline and after ISO stimulation. (**G**) Summary data for CaT frequency (bpm). Summary data in **B** and **G** are shown as violin plots, with symbols within the plots representing individual data points and with median, as well as quartiles, indicated as dashed lines. *P < 0.05, **P < 0.01, and ****P < 0.0001 by 2-way ANOVA with Holm-Sidak multiple-comparison post hoc analyses.

Mice treated with the control constructs exhibited normal surface ECG before and after ISO stimulation. In contrast, mice treated with the *AC_I_*-targeted construct experienced SND before and after ISO injection (Figure 3A), with sinus bradycardia and sinus arrhythmias, as well as a blunted response to β-AR stimulation (Figure 3B). SAN tissues were then excised (Figure 3C), and the mCherry signal was used to validate the successful delivery of the construct using Light Sheet fluorescence microscopy. Green fluorescence signals from HCN4-GCaMP8 transgenic mice was used as the marker for the SAN region, showing that the SAN region was successfully transfected in the merged image (Figure 3D). SAN-specific *AC_I_* gene silenced mice showed abnormal Ca^2+^ signaling with irregular and reduced beating rates (Figure 3, E–G). In summary, *AC_I_^–/–^* mice demonstrate normal cardiac systolic and diastolic function, but they exhibit significant SND with sinus bradycardia and blunted responses to β-AR stimulation.

### No evidence of SND or cardiac dysfunction in AC_VIII_^–/–^ mice.

We further tested the functional roles of another Ca^2+^-activated isoform, AC_VIII_, by taking advantage of *AC_VIII_^–/–^* mice (Supplemental Figures 4 and 8). Echocardiography was performed to quantify cardiac dimensions and function in *AC_VIII_^–/–^* mice compared with WT animals (Supplemental Figure 8). There were no significant differences in HR, fractional shortening (FS), or E/A ratios in *AC_VIII_^–/–^* mice compared with WT animals (Supplemental Figure 8, A–D). In contrast to *AC_I_^–/–^* mice, ambulatory ECG recordings showed normal HR responses to ISO with normal diurnal variations, HR variability, and histograms in *AC_VIII_^–/–^* compared with WT mice (Supplemental Figure 8, E–I). There were no significant differences in intrinsic HR after ANS blockade between the 2 groups (Supplemental Figure 8G). In summary, there was no evidence of cardiac abnormality, SND, or alterations in cardiac function in *AC_VIII_^–/–^* mice (Supplemental Figure 8).

### AC_I_^–/–^ SAN cells exhibit a blunted response of action potential (AP) firing to β-AR stimulation.

**Figure 4:**
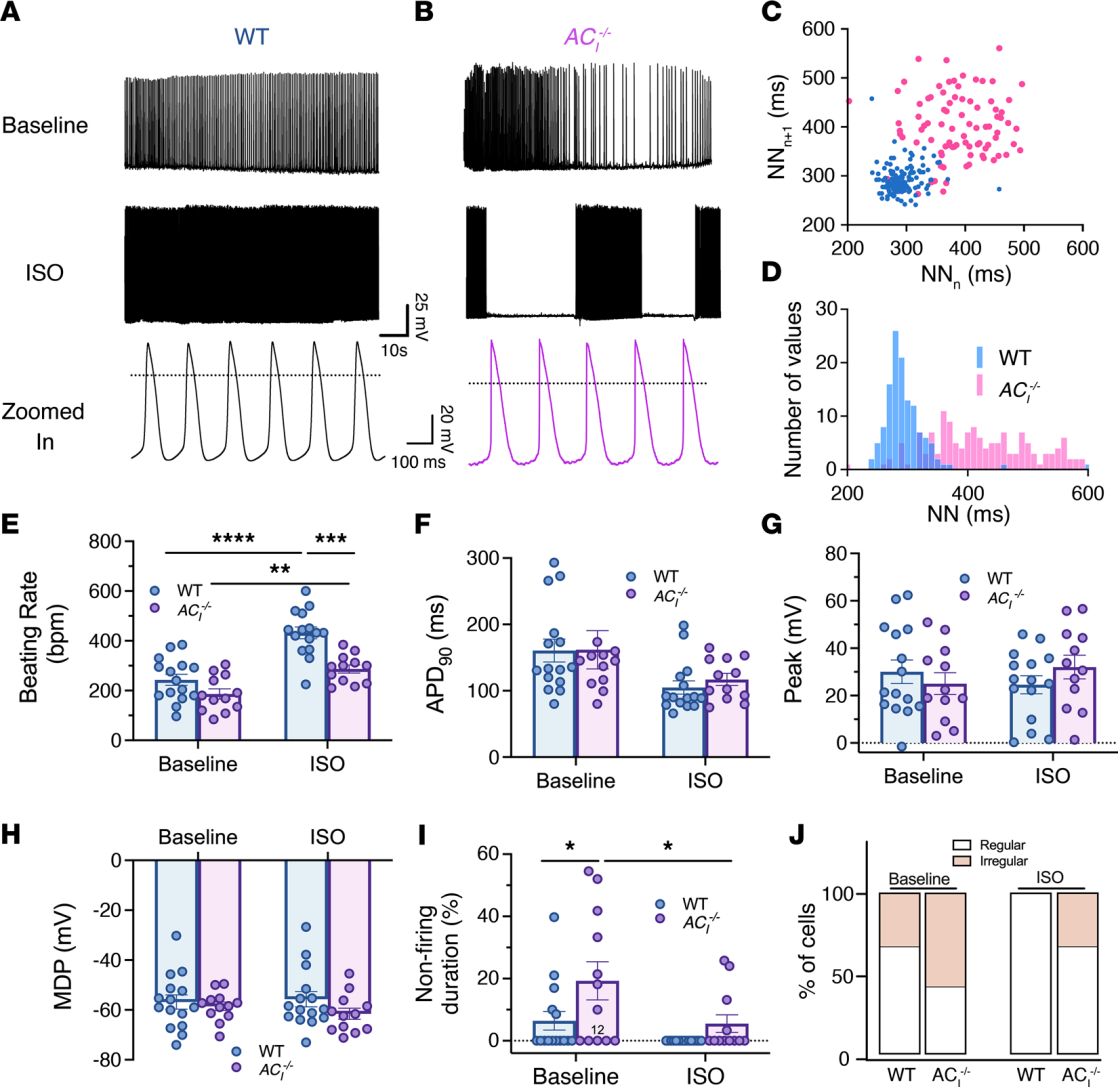
*AC_I_^–/–^* SAN cells exhibit reduced AP firing frequency. (**A** and **B**) Representative traces of spontaneous APs at baseline and after ISO from (**A**) WT and (**B**) *AC_I_^–/–^* SAN cells. Dotted lines in the zoomed-in traces indicate 0 mV. (**C**) Scattered plots of interspike intervals for *n* beat and *n* + 1 beat in ms. (**D**) The number of values for each interspike interval was plotted as histograms. (**E**) Summary data of beating rate (bpm). (**F**) AP duration at 90% repolarization (APD_90_). (**G**) Peak potentials. (**H**) Maximum diastolic potentials. (**I**) Percentages of nonfiring duration. (**J**) Percentages of cells that exhibited nonfiring activity. *n* = 12–15 cells from *n* = 6–7 mice per group. Data are expressed as mean ± SEM. *P < 0.05, **P < 0.01, ***P < 0.001, and ****P < 0.0001 using 2-way ANOVA with Holm-Sidak multiple-comparison post hoc analyses.

To determine the mechanistic underpinnings for sinus bradycardia and SND in *AC_I_^–/–^* mice, APs were recorded from SAN cells isolated from WT and *AC_I_^–/–^* mice using the perforated patch configuration in the current-clamp mode. Under basal conditions, SAN cells isolated from *AC_I_^–/–^* mice exhibited periods of irregularities with bursting activities (Figure 4, A and B). The bursting activities were exacerbated by ISO stimulation. Representative Poincaré plots in Figure 4C demonstrate an increase in interspike variabilities, with longer interspike intervals (lower firing rates) in the histogram plot from *AC_I_^–/–^* compared with that from WT SAN cells (Figure 4D). Quantitatively, the beating rate (quantified from within the burst) was significantly lower in *AC_I_^–/–^* SAN (286.8 ± 16.3 bpm) than in WT SAN cells (432.0 ± 23.7 bpm) after ISO challenge (*P* < 0.001, Figure 4E). The abnormal responses to β-AR are consistent with the ECG findings in vivo. There was no significant difference in AP duration at 90% repolarization (APD_90_; Figure 4F), peak potentials (Figure 4G), or maximum diastolic potentials (Figure 4H). Nonfiring activity was observed in WT SAN cells, as previously described, with periods of regular firing and nonfiring (29–31). However, the percentages of nonfiring duration were significantly increased in *AC_I_^–/–^* SAN (19.3% ± 5.0%) compared with WT SAN (6.4% ± 4.5%) cells (Figure 4I; *P < 0.05). The nonfiring pattern was observed in 33.3% of WT compared with 58.3% of *AC_I_^–/–^* SAN cells. ISO completely abolished the nonfiring mode in WT and reduced the number of cells with nonfiring activity in *AC_I_^–/–^* SAN cells (30%, Figure 4J).

### AC_I_^–/–^ SAN cells show impaired global Ca^2+^ transients (CaTs) with Ca^2+^ alternans and a reduced response of local Ca^2+^ releases (LCR) to β-AR stimulation.

**Figure 5:**
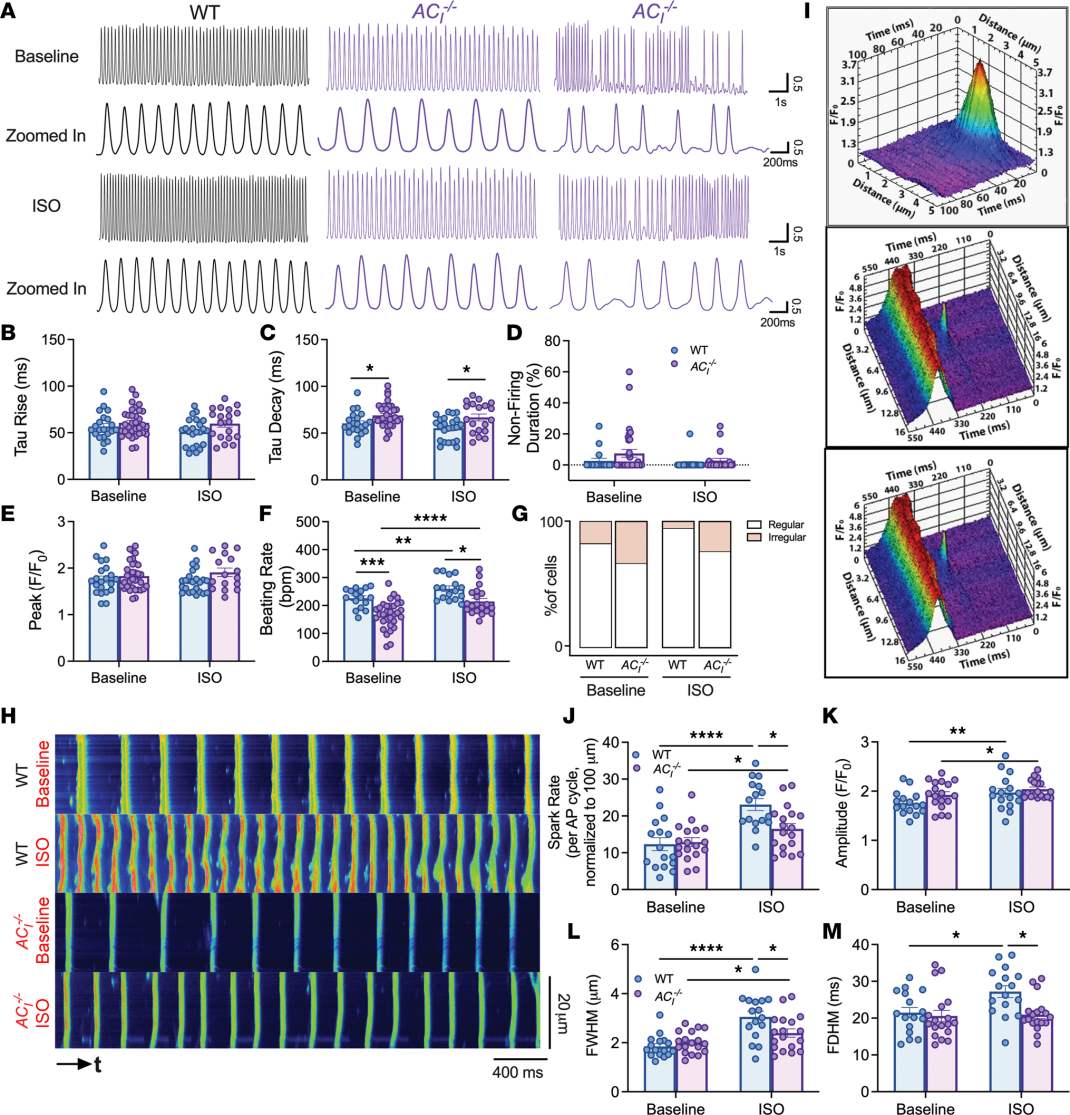
*AC_I_^–/–^* SAN cells exhibit an impaired β-AR response of Ca^2+^ transients (CaTs) with Ca^2+^ alternans and a blunted response of local Ca^2+^ release (LCR) to β-AR stimulation. (**A**) Representative whole-cell CaT traces of SAN cells from WT and *AC_I_^–/–^* mice before and after ISO application. (**B**–**G**) Summary data of τ_rise_ (**B**), τ_decay_ (**C**), percentages of nonfiring duration (**D**), normalized peak amplitude (**E**), beating rate (**F**), and percentage of cells exhibiting nonfiring activity (**G**). Number of symbols in the bar graphs represents number of cells. *n* = 19–33 cells from *n* = 6–7 mice per group. (**H**) Representative LCR recordings from WT and *AC_I_^–/–^* SAN cells before and after ISO application. (**I**) Representative 3D reconstructions of Ca^2+^ sparks in WT SAN cells. (**J**–**M**) Summary data of spark rate (spark numbers per AP cycle, normalized per 100 μm) (**J**), amplitude (**K**), full width at half maximum (FWHM) (**L**), and full duration at half maximum (FDHM) (**M**). Each symbol represents the average of sparks from 1 cell. *n* = 16–18 cells from *n* = 6–7 mice per group. Data are expressed as mean ± SEM. *P < 0.05, **P < 0.01, ***P < 0.001, and ****P < 0.0001 by 2-way ANOVA with repeated measures, followed by Holm-Sidak multiple-comparison post hoc analyses.

Since AC_I_ is a Ca^2+^-activated AC isoform, we examined global CaT and LCR to determine the mechanisms underlying the observed SND in *AC_I_^–/–^* mice. Representative traces of global CaTs for WT and *AC_I_^–/–^* SAN cells are depicted in Figure 5A. *AC_I_^–/–^* SAN cells exhibited evidence of Ca^2+^ alternans both at the basal condition and after ISO application, with beat-to-beat alternations between large and small CaTs for each consecutive beat. Moreover, *AC_I_^–/–^* cells showed irregular firing frequency consistent with the AP and ECG recordings. Although there were no differences in the time constants of the rising phase of CaT (τ_rise_), the time constants of the decay phase of CaT (τ_decay_) was significantly prolonged in *AC_I_^–/–^* compared with WT SAN cells after β-AR stimulation, possibly from a decrease in SR Ca^2+^ uptake (Figure 5, B and C). There was no difference in nonfiring duration (Figure 5D) and peak amplitude (Figure 5E). However, the beating rate was significantly decreased in *AC_I_^–/–^* compared with WT SAN cells at baseline and after ISO application (Figure 5F). Indeed, the percentage of cells that exhibited irregular CaT behavior was higher in *AC_I_^–/–^* SAN cells (Figure 5G).

Analysis of LCRs was performed using IOCBIO Sparks software (32) and a customized interface implemented in LabVIEW to generate 3D reconstructions of Ca^2+^ sparks, as depicted in representative images from WT SAN cells (Figure 5I and Supplemental Figure 9). As expected, spark rate, the amplitude of LCR, full width at half maximum (FWHM), and full duration at half maximum (FDHM) significantly increased with β-AR stimulation in WT SAN cells. LCRs from *AC_I_^–/–^* SAN cells showed a blunted response to β-AR (Figure 5, H–M) with significantly lower spark rates, FWHM, and FDHM after ISO compared with WT SAN cells. The findings are consistent with the colocalization of RyR2 with AC_I_ within the microdomain — consistent with findings in Figure 1, I and J — and support the critical roles of AC_I_ in mediating the enhancement of the Ca^2+^ clock during β-AR stimulation. Nonetheless, a note of caution is warranted in the overall interpretation, since some of the differences between the 2 groups — although statistically significant — are relatively small.

### AC_I_^–/–^ SAN cells demonstrate blunted responses of L-type but not T-type Ca^2+^ currents or Na^+^ currents to β-AR stimulation.

**Figure 6:**
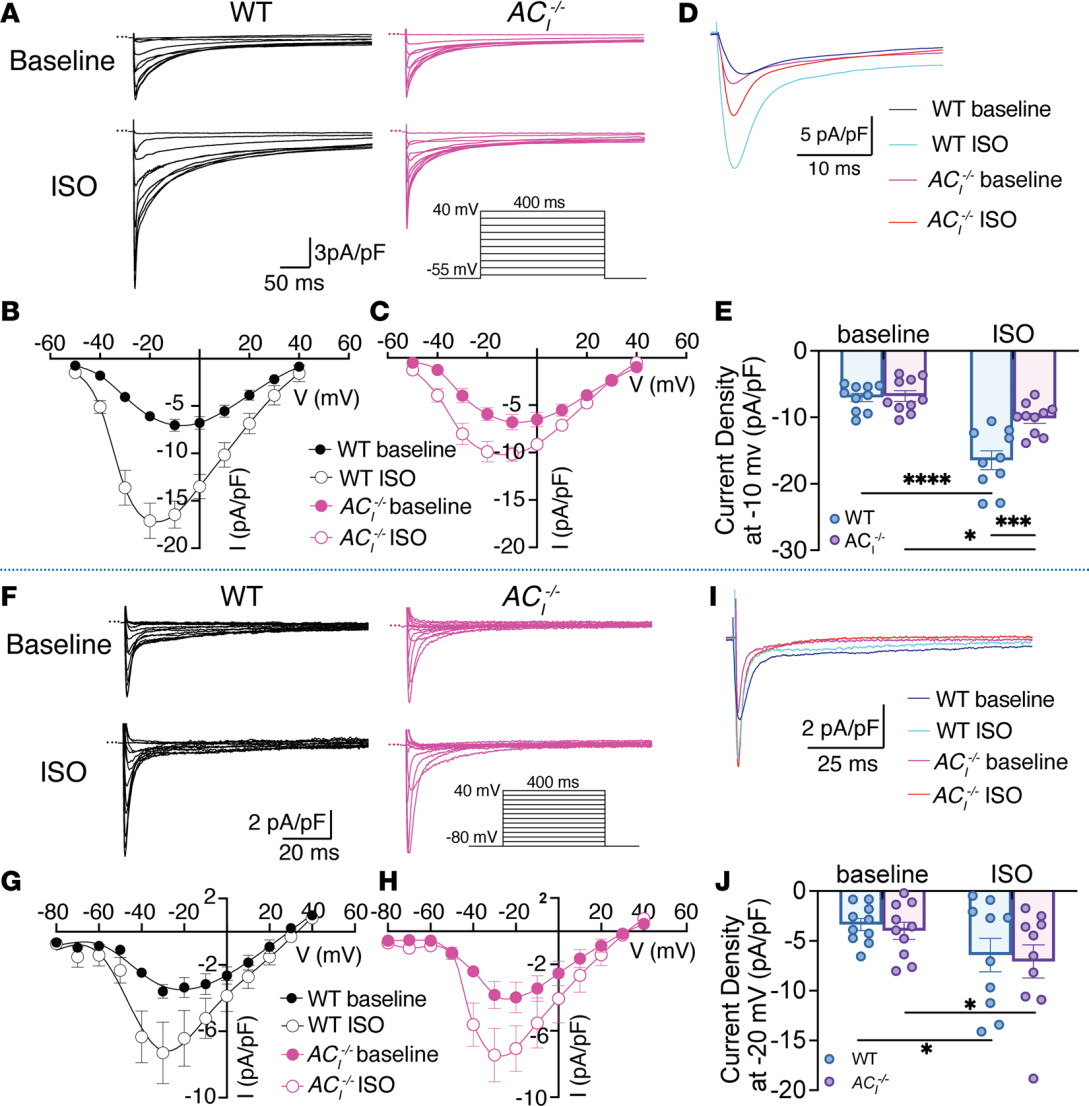
*AC_I_^–/–^* SAN cells demonstrate a significant decrease in β-AR responses of I_Ca,L_ but not I_Ca,T_. (**A**) Representative traces of I_Ca,L_ at baseline (top panels) and after ISO perfusion (bottom panels) in WT (left panels) and *AC_I_^–/–^* SAN cells (right panels). Representative I_Ca_ traces were recorded using 400 ms test pulses from a holding potential of –55 mV to test potentials between –50 and +40 mV with 10 mV increments, using whole-cell patch-clamp mode. (**B** and **C**) Normalized I-V relationship of I_Ca,L_ before and after ISO stimulation in WT (**B**) and *AC_I_^–/–^* (**C**) SAN cells. The normalized conductance-voltage relationship from **B** and **C** are fitted with a Boltzmann function (V_1/2_ are –29.2 and –35.6 mV for WT SAN cells, while V_1/2_ are –30.8 and –33.6 mV for *AC_I_^–/–^* SAN cells, before and after ISO, respectively). (**D**) Superimposition of representative traces for each group at a test potential of –10 mV. (**E**) Summary data of current density at –10 mV. (**F**) Representative traces of I_Ca,T_ at baseline (top panels) and after ISO (bottom panels) in WT (left panels) and *AC_I_^–/–^* (right panels) SAN cells. (**G** and **H**) Normalized I-V relationship of I_Ca,T_ before and after ISO perfusion in WT (**G**) and *AC_I_^–/–^* (**H**) SAN cells. (**I**) Superimposition of representative traces for each group at a test potential of –20 mV. (**J**) Summary data of current density at –20 mV. All currents were normalized to the cell capacitance. *n* = 10 from *n* = 4–5 mice per group. Zero-current levels are shown using dotted lines. Data are expressed as mean ± SEM. *P < 0.05, ***P < 0.001, and ****P < 0.0001 by 2-way ANOVA with repeated measure and Holm-Sidak multiple-comparison post hoc analyses. Results from normality tests are shown in Supplemental Table 1.

β-AR stimulation significantly regulates Ca^2+^ currents (I_Ca_) in the heart. We tested L-type (I_Ca,L_) and T-type (I_Ca,T_) Ca^2+^ currents, at physiological temperature, in response to β-AR stimulation in SAN cells isolated from WT and *AC_I_^–/–^* mice. Representative I_Ca,L_ traces from a holding potential of –55 mV, using external solution without Na^+^ ions, were shown for both groups at baseline and after 1 μM ISO (Figure 6A and Supplemental Table 1). Normalized current-voltage (I-V) relationships at test potentials between –50 to +40 mV of I_Ca,L_ before and after ISO stimulation in WT and *AC_I_^–/–^* SAN cells are shown in Figure 6, B and C. For direct comparison, representative traces at –10 mV are shown for all groups in Figure 6D. The increase in I_Ca,L_ at –10 mV after ISO application was significantly blunted in *AC_I_^–/–^* SAN cells (Figure 6E).

In contrast, ISO significantly increased I_Ca,T_ in both groups relative to the baseline (Figure 6, F–J). Representative I_Ca,T_ traces and I-V curves are shown for both groups at baseline and after ISO (Figure 6, F–H). Representative traces at –20 mV were superimposed (Figure 6I). There were no significant differences in I_Ca,T_ between the 2 groups either before or after ISO application (Figure 6J).

Since Na^+^ current also mediates SAN automaticity, we further tested the roles of Na^+^ current (I_Na_) in SND in *AC_I_^–/–^* mice at physiological temperature. There were no significant differences in I_Na_ in SAN cells from WT and *AC_I_^–/–^* mice under basal condition or after β-AR stimulation. Representative traces of I_Na_, normalized I-V relationship, and summary data are shown (Supplemental Figure 10). The data suggest that AC_I_ regulates L-type but not T-type Ca^2+^ channels or Na^+^ channels in SAN cells. Indeed, high-resolution imaging and PLA support AC_I_’s localization within the microdomain of L-type Ca^2+^ channel, Ca_v_1.2 (Figure 1, I and J).

### AC_I_^–/–^ SAN cells show a significant decrease in the response of I_f_ to β-AR stimulation.

**Figure 7:**
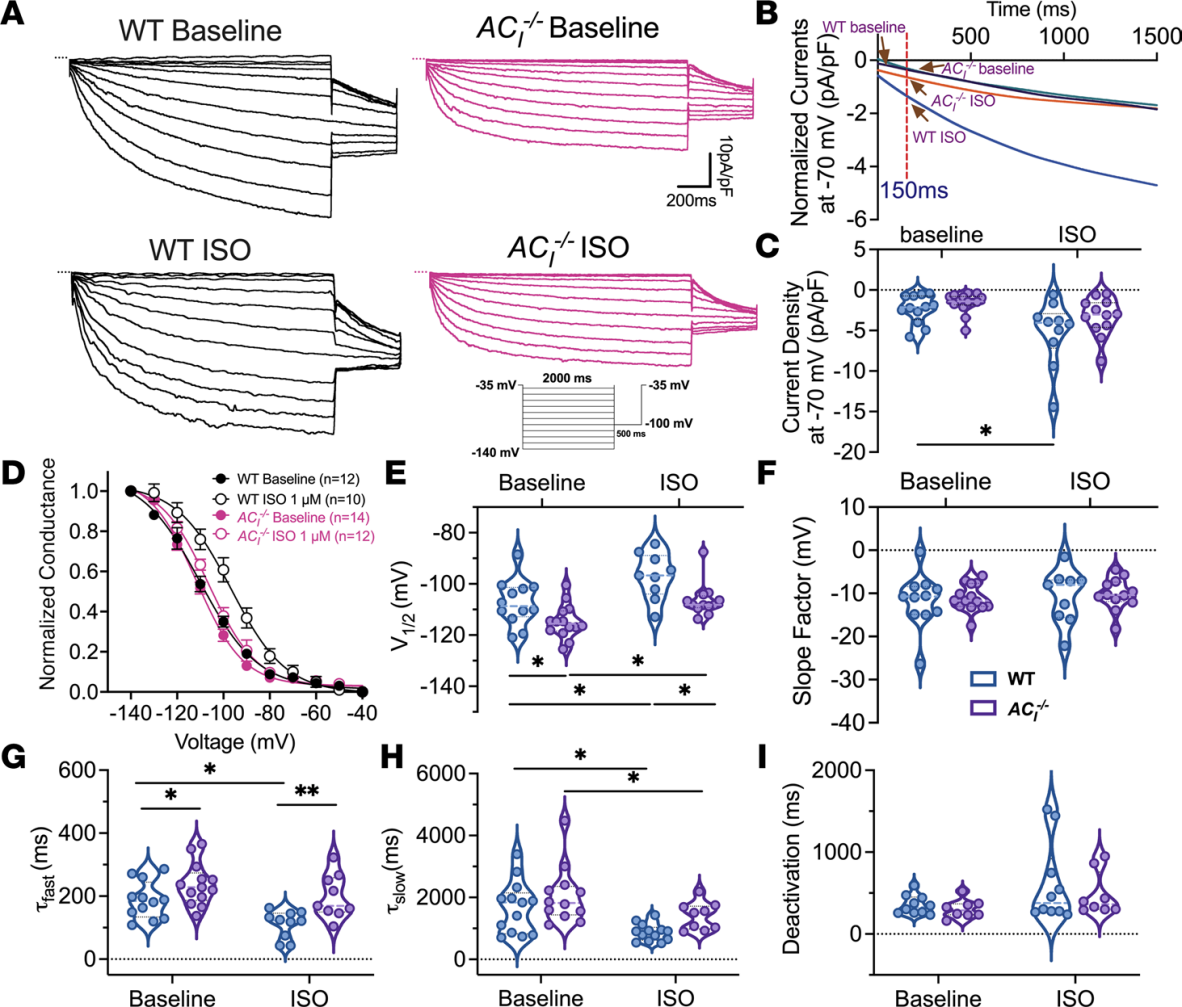
*AC_I_^–/–^* SAN cells show a lack of response of I_f_ to β-AR stimulation. (**A**) Representative traces of I_f_ for WT and *AC_I_^–/–^* SAN cells before and after ISO administration. Zero-current levels are shown using dotted lines. The inset shows a diagram of the voltage-clamp protocol from –140 mV to –40 mV in 10 mV increments from a holding potential of –35 mV. (**B**) Superimposed individual I_f_ traces from each group at –70 mV at baseline and after ISO. (**C**) Summary data of current density at –70 mV. (**D**) Normalized conductance-voltage relationship before and after ISO application, fitted using a Boltzmann function. (**E**–**I**) Summary data for half-activation voltage (V_1/2_) (**E**), slope factor (**F**), τ_fast_ (**G**), τ_slow_ (**H**), and time constants of deactivation (**I**). *n* = 10–14 cells from *n* = 4–5 mice per group. Data are expressed as mean ± SEM in **C** and **E**–**I**. *P < 0.05 and **P < 0.01 by 2-way ANOVA, followed by Holm-Sidak multiple-comparison post hoc analyses. Results from normality tests are shown in Supplemental Table 2.

Funny currents (I_f_), mediated by HCN4 channels, predominantly determine the slope of phase 4 depolarization and modulate spontaneous AP frequency (33). We recorded I_f_ from isolated WT and *AC_I_^–/–^* SAN cells using whole-cell patch-clamp recordings, from –140 to –35 mV in 10 mV increments from a holding potential of –35 mV, at physiological temperature (Figure 7 and Supplemental Table 2). As expected, β-AR stimulation resulted in a significant enhancement of I_f_ in WT SAN cells (Figure 7, A–C) with a significant depolarization shift in the normalized conductance (Figure 7, D and E) and increases in both the fast and slow time constants of activation (τ_fast_, τ_slow_) (Figure 7, G and H). In contrast, β-AR stimulation failed to enhance the amplitude or activation kinetics of I_f_ in *AC_I_^–/–^* SAN cells (Figure 7, A–H), with only a minor shift in normalized conductance (Figure 7, D and E). Specifically, the depolarization shift in the membrane potential or voltage at which the channel is activated by 50% (V_1/2_) is significantly more robust in WT than in *AC_I_^–/–^* SAN cells (Figure 7E, *P < 0.05). There were no differences in the slope factors or deactivation kinetics between WT and *AC_I_^–/–^* SAN cells at baseline or after β-AR stimulation (Figure 7, F and I). The findings provide strong evidence for the functional compartmentalization of AC_I_ and HCN4 channels in SAN cells, consistent with the subcellular colocalization of AC_I_ within the HCN4 microdomain (Figure 1, D–J).

### There are no compensatory changes in the expression levels of HCN4, β_1_-AR, or β_2_-AR in AC_I_^–/–^ SAN.

Western blot analyses of the SAN tissues were performed to directly evaluate possible compensatory changes in the expression of HCN4 or β-AR in *AC_I_^–/–^* mice. There were no significant differences in the protein expression levels of HCN4, β_1_-AR, β_2_-AR, or GPCR kinase 5 (GRK5) (34), which phosphorylates activated GPCRs and promotes β-arrestin binding (Supplemental Figure 11). However, β-arrestin-2, which is involved in GPCR desensitization, was significantly decreased in *AC_I_^–/–^* mice.

### The Ca^2+^-activated AC_I_ isoform is required for the sustained rise in local cAMP after β-AR stimulation in SAN cells.

**Figure 8:**
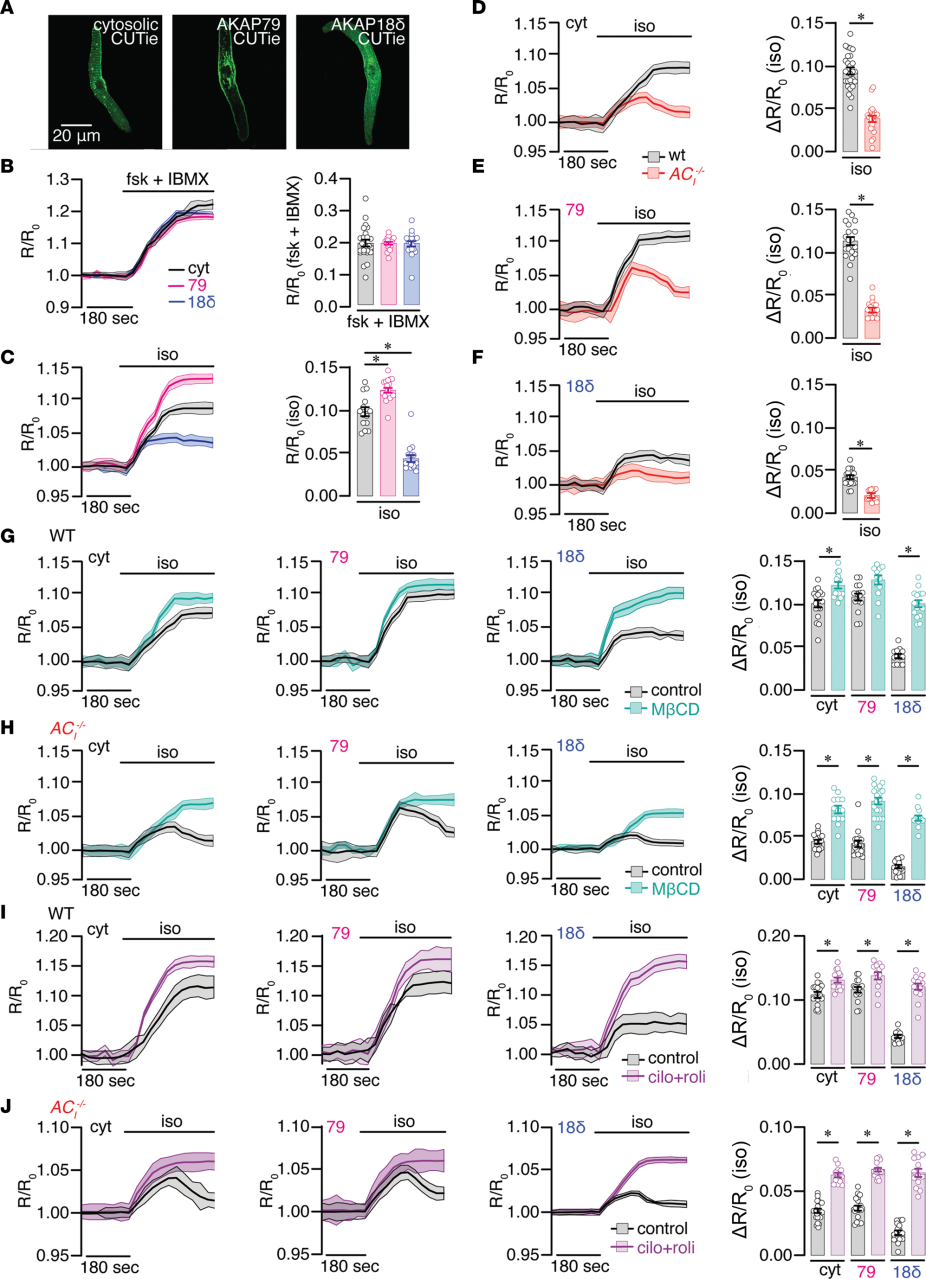
AC_I_ differentially regulates local cAMP signaling at functional microdomains in SAN cells. (**A**) Representative confocal images of SAN cells expressing CUTie sensors localized to the cytosol, plasma membrane (AKAP79-targeted), and SR (AKAP18δ-targeted). (**B** and **C**) Representative time course of changes in FRET response (R/R_0_) in SAN cells expressing CUTie sensors localized to the cytosol (black), plasma membrane (AKAP79; pink) and SR (AKAP18δ; blue) upon application of the AC activator forskolin (10 μM) and the PDE inhibitor 3-isobutyl-1-methylxanthine (IBMX, 100 μM) (**B**), and the β-AR agonist isoproterenol (ISO, 1 μM) (**C**). The FRET ratios were normalized to basal levels before treatment. (**D**–**F**) Time course of changes in the magnitude of normalized FRET responses (R/R_0_) in WT (black) and *AC_I_^–/–^* (red) SAN cells expressing the CUTie sensors after stimulation with ISO (1 μM). Bar graphs on the right show corresponding summary data for the maximal increase in the FRET ratio response to ISO. (**G**–**J**) Representative time course of changes in the magnitude of normalized FRET responses (R/R_0_) in WT (**G** and **I**) and *AC_I_^–/–^* (**H** and **J**) SAN cells expressing CUTie sensors localized to the cytosol (cyt), plasma membrane (AKAP79) and SR (AKAP18δ) upon application of ISO (1 μM) in control (black) and SAN cells treated with 100 μM methyl-β-cyclodextrin (MβCD) (blue, **G** and **H**) or 10 μM cilostamide and 10 μM rolipram (pink, **I** and **J**). Number of symbols in the bar graphs represents number of cells (*n* ≥ 10) from *n* = 4–5 mice (independent SAN isolations). Data are presented as mean ± SEM. *P < 0.05 by Student’s *t* test.

Since the product of AC activation is cAMP, we exploited the latest cAMP fluorescence resonance energy transfer–based (FRET-based) biosensors to elucidate the subcellular mechanism of AC_I_-mediated HR regulation. Single isolated SAN cells were transfected for 36–40 hours with cAMP universal tag for imaging experiments (CUTie) with sensors localized to the cytosol, plasma membrane (AKAP79), and SR (AKAP18δ), as shown in representative confocal images (Figure 8A). The AC activator forskolin (10 μM) and the phosphodiesterase (PDE) inhibitor 3-isobutyl-1-methylxanthine (IBMX, 100 μM) were used to determine the maximal cAMP responses in the 3 specific regions. Figure 8B and Supplemental Figure 12 show that SAN cells exposed to forskolin and IBMX exhibit similar maximal responses in the normalized FRET signal (R/R_0_), which facilitates comparison of cAMP signal in different subcellular domains. ISO applied to WT SAN cells expressing the cytosolic, membrane or SR CUTie sensors revealed distinctive cAMP production in the different subcellular domains (Figure 8C). The membrane signal was the highest, followed by cytosolic, and finally the SR.

In WT SAN cells expressing the cytosolic CUTie sensor, ISO induced a dose-dependent production of cAMP with an EC_50_ of 0.51 ± 0.23 nM (Supplemental Figure 12, F and I). Genetic ablation of *AC_I_* rightward shifted the EC_50_ of ISO-induced cAMP to 224 ± 0.16 nM (Supplemental Figure 12, G and I; ***P < 0.001). In contrast, there was no significant change in EC_50_ of ISO-induced cAMP in *AC_VIII_^–/–^* SAN cells (0.87 ± 0.32 nM; Supplemental Figure 12, H and I). Further experiments examining the time-dependent changes in cAMP after β-AR stimulation found that the localized increase in cAMP in the cytosol, at the membrane, and at the SR was significantly blunted in *AC_I_^–/–^* compared with WT SAN cells (Figure 8, D–F). Notably, in *AC_I_^–/–^* SAN cells, there was an initial rise followed by a decay over time after β-AR stimulation, suggesting a lack of sustained AC_I_-dependent response. Collectively, the data support the critical roles of Ca^2+^-activated isoform of AC in maintaining the sustained rise in local cAMP required for HR responses to β-AR stimulation.

Next, we pretreated WT (Figure 8G) and *AC_I_^–/–^* SAN cells (Figure 8H) with methyl-β-cyclodextrin (MβCD, 100 μM), a cholesterol remover and caveolar disruptor, after transfection with the 3 biosensors. MβCD did not significantly alter local cAMP levels at the membrane region but caused a significant increase in cAMP production in the cytosol and the SR region in WT SAN cells after β-AR stimulation. In contrast, MβCD pretreatment in *AC_I_^–/–^* SAN cells significantly negated the decay in cAMP levels in the cytosol, at the membrane and the SR regions. Simultaneous pretreatment with cilostamide and rolipram (cilo+roli, 10 μM each) — which are PDE3 and PDE4 inhibitors, respectively — produced similar effects to cAMP levels as MβCD in *AC_I_^–/–^* SAN cells (Figure 8, I and J). The results were not observed when PDE2, PDE3, or PDE4 inhibitors were applied alone (Supplemental Figure 13). These results suggest compartmentalization of multiple PDEs and/or their signaling partners via caveolae scaffolding is necessary for regulating local cAMP levels (35, 36).

### AC_I_ is the main Ca^2+^-activated isoform in SAN cells.

Additionally, to identify possible Ca^2+^ sources for AC_I_ regulation, we quantified cAMP levels in the presence of 1 μM nifedipine, 1 μM ryanodine, or the combination (Supplemental Figure 14). WT SAN cells subjected to both nifedipine and ryanodine demonstrated a significant decrease in cAMP level, relative to control SAN cells. In contrast, inhibition of I_Ca,L_ and SR Ca^2+^ release did not significantly alter cAMP level in *AC_I_^–/–^* SAN cells. These results suggest that AC_I_ is the main Ca^2+^-activated isoform in SAN cells and that the Ca^2+^ that regulates AC_I_ is derived from both Ca^2+^ entry via L-type Ca^2+^ channels and SR Ca^2+^ release.

## Discussion

There are current gaps in our knowledge regarding the dominant AC isoforms and the specific roles of Ca^2+^-activated ACs in the SAN. We, therefore, took advantage of SAN-specific CRISPR/Cas9 *AC_I_*-targeted gene silencing mice, as well as *AC_I_^–/–^* and *AC_VIII_^–/–^* mice, to determine the functional significance of the 2 Ca^2+^-activated isoforms, AC_I_ and AC_VIII_, in SAN automaticity. The significance of our study stems from findings utilizing an array of complementary techniques. In contrast to atrial and ventricular myocytes, we identify AC_I_ as the predominant Ca^2+^-activated isoform, mediating cAMP signaling in SAN that resides within a functional microdomain with Cav-3, HCN4, Ca_v_1.2, and RyR2. Global or SAN-specific KO of *AC_I_* results in SND and a blunted HR response to β-AR stimulation. With pharmacological blockade of the ANS in vivo, *AC_I_^–/–^* mice show a lower intrinsic HR. Local cAMP in *AC_I_^–/–^* SAN cells shows an initial rise followed by a decay over time after β-AR stimulation. The data support AC_I_’s critical role in mediating the sustained rise in SAN automaticity in response to β-AR.

### The unique roles of the Ca^2+^-activated AC isoform in SAN function.

cAMP is a critical second messenger that regulates cardiac contractility and chronotropy via activities of cAMP-responsive ion channels and pumps (37, 38). The impact, however, depends on a multitude of factors, including the relative expression of the predominant AC isoforms, as well as their compartmentalization with effector proteins. In contrast to atrial and ventricular myocytes that express mainly AC_V_ and AC_VI_ isoforms (16), we demonstrate diverse AC expression at the transcript and protein levels in mouse SAN cells, with AC_I_ and AC_VI_ as the predominant isoforms, followed by AC_III_, AC_IV_, and AC_VII_ (Figure 1). The relative abundance and differential expression of the AC_I_ isoform in SAN compared with ventricular and atrial myocytes suggest a critical role of AC_I_ in the SAN function. AC_I_ is known to be critically involved in learning and memory formation (39). Moreover, AC_I_-deficient mice were reported to have disrupted retinotopic ordering (40, 41). Indeed, SAN cells show higher basal activities of cAMP and protein kinase A (PKA) than ventricular myocytes (42). AC_I_, which is a Ca^2+^-activated AC isoform, likely contributes to the higher basal level of cAMP (43).

### Synergism among different Ca^2+^-activated AC isoforms.

Synergism among different Ca^2+^-activated AC isoforms have been described in multiple cell types (44). AC_VIII_ is another Ca^2+^-activated isoform that has been extensively studies in the heart (34, 45–49). Previous studies have used cardiac-specific overexpression of *AC_VIII_* transgenic mice and found that overexpression of *AC_VIII_* in SAN markedly impacts HR and rhythm in the transgenic mice. In contrast, our current study utilized *AC_VIII_*^–/–^ mice. We did not observe changes in basal HR, intrinsic HR, or responses to ISO in the *AC_VIII_*^–/–^ mice compared with the WT animals (Supplemental Figure 8). Additionally, single-cell reverse transcription PCR (RT-PCR) showed very low transcript expression of AC_VIII_ in both *AC_I_*^–/–^ mice and WT animals (Figure 1A and Supplemental Figure 1A).

### AC_I_’s role in SAN automaticity.

Recent evidence suggests that both membrane and Ca^2+^ clocks jointly regulate SAN automaticity (50), and ACs significantly contribute to the coupled clock (43). Our current study demonstrates that *AC_I_* ablation significantly blunted β-AR modulation of SAN automaticity, due to the lack of sustained rise in local cAMP (Figure 8). Mechanistically, KO of *AC_I_* significantly impairs β-AR stimulation of LCRs (Figure 5), I_Ca,L_ (Figure 6), I_f_ (Figure 7), and AC-dependent cAMP signaling (Figure 8), leading to irregularity in AP firing with significant periods of AP cessations. The bursting activities of the spontaneous APs observed in the KO mice are reminiscent of the behaviors observed in the previously reported *NCX*-KO mice (51). A decrease in NCX function results in a gradual accumulation of local Ca^2+^ concentration and an increase in the activation of small-conductance Ca^2+^-activated K^+^ (SK) currents, documented to be expressed in pacemaking cells (51), leading to periods of cessation of firing activities.

Additionally, CaTs show evidence of Ca^2+^ alternans with a significant increase in τ_decay_ in *AC_I_^–/–^* SAN cells. The findings are consistent with a significant decrease in SR Ca^2+^ reuptake by SR Ca^2+^-ATPase, as recently demonstrated in ventricular myocytes (52). KO of *AC_I_* in SAN cells is expected to decrease cAMP-mediated, PKA-dependent phosphorylation of SR proteins, including phospholamban, which may represent one of the mechanisms for the increased τ_decay_ and Ca^2+^ alternans.

### Functional compartmentalization of AC_I_ in the SAN.

Synchronization of the coupled clock in the SAN is restricted to precise subcellular microdomains with discrete clusters of ion channels, transporters, and regulatory receptors working in concert (53). Caveolin serves as the scaffolding protein to compartmentalize specialized proteins to initiate diverse molecular signaling (54). Our group has previously demonstrated that AC_VI_ is localized in the plasma membrane outside the T-tubule in ventricular myocytes (17). In contrast, AC_V_ is localized mainly in the T-tubular region, and the direct protein-to-protein interaction between Cav-3 with AC_V_ and PDEs is responsible for the compartmentalization of AC_V_ signaling (17). Ca^2+^-activated AC_I_ and AC_VIII_ are localized within lipid raft microdomains in the SAN (19). Our current study shows colocalization of AC_I_ and Cav-3, as well as HCN4, Ca_v_1.2, and RyR2, within microdomains of < 40 nm based on PLA (Figure 1). The colocalization was further supported using functional analyses, showing a significantly blunted response of HR, LCRs, I_f_, and I_Ca,L_ to β-AR stimulation in *AC_I_^–/–^* SAN cells. In contrast, there were no significant differences in basal current or after β-AR stimulation of I_Ca,T_ and I_Na_ between WT and *AC_I_^–/–^* SAN cells. It is important to note that controversy remains regarding responses of I_Ca,T_ to β-AR stimulation. While some studies found minor effects in I_Ca,T_ after β-AR stimulation (55–57), others demonstrated significant regulation by β-AR (58).

Further analyses demonstrate that the effects of *AC_I_* ablation on β-AR modulation are distinct among the 3 downstream targets in the coupled clock — LCRs, I_Ca,L_, and I_f_. Specifically, *AC_I_* KO significantly negated the enhancement of I_f_, with no significant changes in current density or activation kinetics of I_f_ after β-AR stimulation with only a minor shift in V_1/2_ in *AC_I_^–/–^* SAN cells, suggesting that HCN4 channels may form compartmentalization with AC_I_ almost exclusively. Our findings are consistent with the previously published data in the *AC_I_* and *AC_VIII_*–double KO mice, supporting the regulation of I_f_ by Ca^2+^-activated AC isoforms (59).

In contrast, Ca^2+^ channels (possibly different isoforms of Ca^2+^ channels) and RyR2 may form functional units with distinct isoforms of ACs, including AC_I_. We have previously shown the functional expression of both Ca_v_1.2 and Ca_v_1.3 L-type Ca^2+^ channels in SAN (60). Future studies are required to decipher the functional significance of compartmentalization of different isoforms of Ca^2+^ channels with ACs.

### Critical roles of AC_I_ in the sustained rise of local cAMP under β-AR stimulation.

To directly quantify local cAMP levels, we used FRET-based cAMP biosensors localized to the cytosol, at the plasma membrane or the SR region. The local cAMP responses to β-AR stimulation are significantly blunted in all 3 regions in *AC_I_^–/–^* SAN cells (Figure 8). There is an initial rise in local cAMP levels with β-AR stimulation from other AC isoforms. However, the effects of β-AR stimulation fail to sustain over time, suggesting AC_I_’s critical roles in sustaining local cAMP in the cytosol, at the plasma membrane and the SR region.

The effects of MβCD on removing cholesterol and disrupting lipid rafts and caveolar domains (61) in WT SAN cells are mirrored by the synergistic effects of PDE3 and PDE4 inhibitors, with a pronounced enhancement of local cAMP at the SR (Figure 8). The findings suggest that AC_I_’s actions at these functional microdomains are balanced and modulated by the localized effects of PDE3 and PDE4 that degrade cAMP. In *AC_I_^–/–^* SAN cells, the lack of a sustained rise in the local cAMP by β-AR stimulation is negated by MβCD or the combination of PDE3 and PDE4 inhibitors, suggesting compartmentalization of other AC isoforms with PDE3 and PDE4.

In conclusion, SAN cells express a diverse repertoire of ACs with AC_I_ as the predominant Ca^2+^-activated isoform. The diversity of ACs in SAN cells likely provides the needed safety factor for the critical pacemaking activities in the heart. AC_I_ isoform plays exclusive roles in the chronotropic regulation of the heart with no discernable actions on cardiac systolic or diastolic function. Genetic ablation of *AC_I_* results in SND in vivo and impaired SAN automaticity in vitro. Due to its central characteristic as a Ca^2+^-activated isoform, AC_I_ provides a unique role in the sustained rise of local cAMP during β-AR stimulation. HCN4 channels of the coupled clock form functional microdomains almost exclusively with AC_I_, while L-type Ca^2+^ channels or different isoforms of L-type Ca^2+^ channels and RyR2 likely form compartmentalization with different AC isoforms. Collectively, our data support functional microdomains of AC_I_ with important Ca^2+^ handling proteins and HCN4 channels that play critical roles in sustaining the rise of local cAMP under β-AR stimulation (Figure 8).

## Methods

Supplemental Methods are available online with this article.

### Animal models.

Male and female WT, *AC_I_^–/–^*, and *AC_VIII_^–/–^* mice (21, 22) 10–15 weeks old in the C57BL6/J background were used. Mice were housed individually in a 12-hour light/12-hour dark environment. All experiments were performed in a blinded fashion, with different investigators conducting animal handlings, cardiomyocyte isolations, data collection, and analyses.

### SAN cell isolation.

SAN cells were isolated as described (62–66).

### Single-cell qPCR.

Single cells were identified and isolated with patch pipettes under a microscope. RNA was isolated from single cells using Single Cell-to-CT qPCR Kit (Thermo Fisher Scientific). Single-strand cDNA was synthesized using Superscript III. The qPCR was performed using predesigned TaqMan Gene Expression assays probes (Thermo Fisher Scientific).

### smFISH.

SmFISH was performed as described (67) in WT and *AC_I_* KO SAN sections using probes for AC_I_, AC_V_, and AC_VI_.

### Whole-mount IHC.

Whole-mount IHC was performed as described previously (30, 68). The following primary antibodies were used: (a) anti-HCN4 (Abcam, ab66501, 1:200 dilution), a polyclonal antibody raised against rat HCN4, and (b) anti-AC_I_ (Santa Cruz Biotechnology Inc., sc-365350, 1:100 dilution), a monoclonal antibody raised against mouse AC_I_. SAN tissue was washed with PBS (3 × 10 minutes) and then incubated with anti-rat and anti-mouse secondary antibodies (Jackson ImmunoResearch Laboratories, 1:1,000 dilution) for 4 hours at room temperature in the dark. It was then washed in PBS (3 × 10 minutes) and incubated for 2 hours with 20% DMSO diluted in PBS. Coverslips were mounted on the slides with ProLong Diamond Antifade Mountant (Thermo Fisher Scientific). The slides were sequentially imaged using a Zeiss 900 confocal laser-scanning microscope equipped with an Airyscan detector module, a Plan-Apo 63× 1.4 NA oil-immersion objective, and 488/561 lasers. Imaris software (Bitplane) was used to perform 3D reconstructions.

### Echocardiography.

Echocardiography to assess systolic and diastolic function were performed using Vevo 2100 (VisualSonics, Fujifilm) imaging system and a MS 550D probe (22–55 MHz) (69, 70).

### Hemodynamic monitoring.

Hemodynamic monitoring was performed as previously described (67).

### ECG telemetry.

All telemetry placements were performed 1 week before the start of each experiment. Mice were anesthetized with ketamine (80 mg/kg) and xylazine (5 mg/kg) before placement of a transmitter (Data Sciences International [DSI]) into the abdominal cavity with s.c. electrodes in the lead I configuration. Baseline measurements were recorded for 24 hours and followed by i.p. injection of ISO (0.1 mg/kg, i.p.) in *AC_I_^–/–^*, *AC_VIII_^–/–^* and WT animals. Atropine (2 mg/kg, i.p.) and propranolol (1 mg/kg, i.p.) were used to block the heart’s autonomic control. The analog telemetric ECG signals were digitized at 1 kHz and recorded using Ponemah software (DSI). R peaks of the ECG signal were detected, and the mean HR was calculated from the RR-I and averaged for 1 minute. For baseline recordings, *t* = 0 corresponds to noon, while *t* = 24 corresponds to midnight. HR variability (HRV) was plotted as RR-I against the next RR-I.

### SAN-specific CRISPR/Cas9-mediated gene silencing of AC_I_.

A transgenic mouse model expressing a fluorescent Ca^2+^ indicator (GCaMP8) under the control of the *Hcn4* promoter was previously generated and used for the study(71). CRISPR/Cas9 system containing 3× sgRNA (GeneCopoeia) was used to specifically target the AC_I_ isoform, followed by in vivo delivery using liposome and SAN painting technique (27, 28). A vector containing a scrambled sequence was used as control. Both the targeting and control vectors contained mCherry and were encapsulated in liposomes. The liposomal emulsion was delivered onto the SAN region under direct visualization. ECG and echocardiograms were performed 5–7 days after surgery at baseline and after ISO injection. Light Sheet-Based Fluorescence Microscopy (LSFM) was performed in freshly dissected SAN to confirm that the in vivo gene delivery was successful. Green fluorescence protein (GFP) and mCherry signals were simultaneously detected during live SAN imaging.

### LSFM.

Freshly dissected tissues were placed in normal Tyrode’s solution, immersed in 1.5% agarose in a capillary tube, and mounted inside the Lattice Lightsheet 7 microscope (Carl Zeiss). During experiments, tissue was maintained at 37°C and constantly gassed with 95% O_2_/5% CO_2_. Baseline measurements were taken before the application of 1 μM of ISO. Imaris software (Bitplane) was used to perform 3D reconstructions.

### Immunofluorescence confocal microscopy.

Immunofluorescence labeling was performed as previously described (17). The following primary antibodies were used to incubate the cells overnight at 4°C: (a) anti-HCN4 (Abcam, 1:300 dilution), a polyclonal antibody raised against rat HCN4; (b) anti-AC_I_ (Santa Cruz Biotechnology, 1:100 dilution), a monoclonal antibody raised against mouse AC_I_; and (c) anti–Cav-3 (1:300, Thermo Fisher Scientific), a polyclonal antibody raised against rabbit Cav-3. Cells were washed with PBS (3 × 10 minutes) and then incubated with anti-rat, anti-mouse, or anti-rabbit secondary antibodies (Jackson ImmunoResearch Laboratories, 1:500 dilution) for 1 hour at room temperature. Control experiments performed by incubation with secondary antibody only did not show positive staining under the same experimental conditions. Identical settings were used for all specimens.

STED microscopy was performed on a Leica STED (TCS SP8 STED 3×) microscope with an HC PL APO 100×/1.4 NA STED objective in STED mode (Leica Microsystems). Using Huygens professional software, deconvolution was limited to 15 iterations and a signal/noise ratio of 4 with a manual evaluation of background intensity.

### Electrophysiology.

Whole-cell L-type and T-type Ca^2+^ currents (I_Ca,L_ and I_Ca,T_), HCN currents (I_f_) and Na^+^ current (I_Na_) were recorded at 36°C ± 0.5°C using conventional whole-cell patch-clamp techniques (72). Current-voltage relations were assessed before and after the application of ISO (1 μM). Cell capacitance was calculated as the ratio of total charge (the integrated area under the current transient) to the magnitude of the pulse (20 mV). Currents were normalized to cell capacitance to obtain the current density. The series resistance was compensated electronically. In all experiments, a series resistance compensation of ≥ 85% was obtained. The currents and membrane potentials were recorded using Axopatch 200A amplifier and Digidata 1440 digitizer (Molecular Devices). The signals were filtered at 2 kHz using a 4-pole Bessel filter, digitized at a sampling frequency of 10 kHz for I_Ca_ and I_Na_, filtered at 1 kHz, and digitized at a sampling frequency of 5 kHz for I_f_. All experiments were performed using 3M KCl agar bridges connecting the ground electrode to the recording chamber. Borosilicate glass electrodes were pulled with a P-97 micropipette puller (Sutter Instruments). The resistance of the electrodes was ~2–3 MΩ when filled with the pipette solutions. Data acquisition and analysis were carried out using pClamp 10 software (Molecular Devices) and Origin Software (OriginLab). No leak compensation was used for the recordings. Recordings were obtained from cells with seal resistance of 1–5 GΩ. Cells with seal resistance less than 1 GΩ were rejected.

Spontaneous APs and AP firing frequencies in single SAN cells were recorded using the perforated patch-clamp technique at 36°C ± 0.5°C. For AP recordings, amphotericin B (240 μg/mL) was added into the pipette solution. Spontaneous APs were recorded in Tyrode’s solution containing (in mM): 140 NaCl, 5.0 HEPES, 5.5 glucose, 5.4 KCl, 1.8 CaCl_2_, and 1.0 MgCl_2_ (pH 7.4). The pipette solution contained (in mM): 130 potassium aspartate, 10 NaCl, 10 HEPES, 0.04 CaCl_2_, 2.0 Mg-ATP, 7.0 phosphocreatine, and 0.1 Na-GTP, with pH adjusted to 7.2 with KOH. All the chemicals were purchased from Sigma-Aldrich unless specified.

### PLA.

Colocalization between AC_I_ and Cav-3, AC_I_ and HCN4, AC_I_ and Ca_v_1.2, AC_I_ and RyR2, AC_I_ and β_1_-AR, and AC_I_ and β_2_-AR were detected by a Duolink In Situ PLA kit (Sigma-Aldrich) (73).

### Whole-cell CaT measurements.

IonOptix contraction system was used to detect spontaneous CaTs from single isolated SAN cells. Freshly isolated SAN cells were loaded with 5 μM Fluo-4 AM (F14201, Thermo Fisher Scientific) for 15 minutes at room temperature. Cells were then perfused with Tyrode’s solution (36°C ± 0.5°C) continuously. Baseline measurements were taken before ISO was applied in both WT and *AC_I_^–/–^* mice. The maximal Fluo-4 fluorescence was measured at peak amplitude and was normalized to the average of baseline fluorescence (F_0_). Background fluorescence was subtracted for each recording.

### LCR and CaT detection via confocal line scanning.

LCR and CaTs were quantified as previously described (74).

### Culture of SAN cells.

SAN cells were first isolated as described above and maintained in culture as we have previously described (66) (Supplemental Figure 12, A–C). We demonstrate that SAN cells maintained in our culture condition retain their elongated morphology and AP waveform for up to 40 hours. The culturing condition does not change β-adrenergic–mediated cAMP signal as determined in freshly dissociated and cultured SAN cells from a cardiac-specific cAMP reporter mouse (66).

### Adenoviral transfection of cAMP biosensors in SAN cells and confocal imaging.

For adenoviral transfection, the media was replaced with 500 μL of serum-free medium containing adenoviral vectors carrying different versions of the FRET-based CUTie sensor (75). Accordingly, we employed the cytosolic CUTie, the membrane-targeted AKAP79-CUTie, and SR-targeted AKAP18δ-CUTie. Cells infected with the desired adenoviral vectors were incubated at 37°C with 5% CO_2_ for 36–40 hours. Adenoviral vectors were produced using the AdEasy system (Qbiogene Inc.) (76). A Zeiss LSM 700 laser scanning confocal microscope paired with a Zeiss 63× oil immersion lens (numerical aperture = 1.4) was used to collect images at different optical planes (*z* axis steps: 0.4 μm) of the yellow fluorescent protein (YFP) fluorescence associated with each FRET construct to confirm expression and expected localization.

### FRET imaging and quantification.

Glass coverslips with SAN cells were transferred to a glass-bottom culture dish (MatTek) containing 3 mL PBS at room temperature. A Leica DMI3000B inverted fluorescence microscope (Leica Biosystems) equipped with a Hamamatsu Orca-Flash 4.0 digital camera controlled by Metaflor software (Molecular Devices) acquired phase contrast, cyan fluorescent protein (CFP), and YFP images. Phase contrast and CFP480 images were collected with 20× and 40× oil immersion objective lenses, while YFP images were collected using only the 40× oil immersion objective lens. Images for FRET analysis were recorded by exciting the donor fluorophore at 430–455 nm and measuring emission fluorescence with 2 filters (475DF40 for cyan and 535DF25 for yellow). Images were subjected to background subtraction and acquired every 30 seconds with exposure time of 200 ms for each channel. The donor/acceptor FRET ratio was calculated and normalized to the ratio value of baseline before ISO. Averages of normalized curves and maximal response to stimulation were graphed based on FRET ratio changes. The binding of cAMP to each FRET biosensor increased the ratio of YFP/CFP and was interpreted as an increase in cAMP levels. Experiments were performed at room temperature.

### Western blot.

SAN tissue from WT and *AC_I_^–/–^* mice were flash frozen in liquid nitrogen for Western blotting experiments. The same amount of total protein (5 μg) was loaded in each lane. Membranes were blocked in 3% nonfat dry milk (Bio-Rad) in TBST for 1 hour (room temperature) and then incubated with primary antibodies including anti-HCN4 (1:500 dilution, APC-052, Alomone Labs), anti–β_1_-AR (1:1,000 dilution, PA1-049, Thermo Fisher Scientific), anti–β2-AR (1:1,000, PA5-27083, Thermo Fisher Scientific), anti–GRK-5 (1:1,000 dilution, PA5-23189, Thermo Fisher Scientific), anti–β-arrestin-2 (1:1,000 dilution, PA1-732, Thermo Fisher Scientific), and anti-GAPDH (1:5,000, ab8245, Abcam) antibodies, all in 3% nonfat dry milk in TBST overnight at 4°C. On the next day, the membranes were incubated with conjugated secondary antibody (Abcam) for 1 hour at room temperature, and the bands were visualized using Fujifilm LAS-3000 Imager.

### Chemicals.

All chemicals were purchased from Sigma-Aldrich unless indicated otherwise. Laminin (catalog 23017015) was obtained from Invitrogen, blebbistatin (catalog 13013) was obtained from Cayman Chemical, and IBMX (catalog 2845) was obtained from Tocris Bioscience.

### Data availability.

All data generated or analyzed in this study are included in the main manuscript and/or supplemental figures. Raw data of images are available upon request. Source data are provided with this paper.

### Statistics.

Data were analyzed using GraphPad Prism software and presented as mean ± SEM. Data were assessed for potential outliers using the GraphPad Prism Outlier Test and for normality of distribution. Statistical significance was then determined using appropriate unpaired 2-tailed Student’s *t* test, nonparametric tests, 1-way ANOVA, or 2-way ANOVA for multiple comparisons with appropriate post hoc test. Two-way ANOVA was followed by a Holm-Sidak multiple-comparison test. General linear model was used for 2-way repeated measures, and mixed-effect model was used when there were missing values. *P* < 0.05 was considered statistically significant.

### Study approval.

The present investigation conforms to the *Guide for the Care and Use of Laboratory Animals* (National Academies Press, 2011) and was performed in accordance with the protocols and guidelines approved by the IACUC of UCD.

## Author contributions

LR, PNT, ENY, MFN, and NC designed the research; LR, PNT, RRG, VT, HAL, SP, and XDZ performed experiments; LR, PNT, RRG, XDZ, ENY, MFN, and NC analyzed data; LR, PNT, RRG, XDZ, ENY, MFN, and NC wrote the manuscript; CMM, LFS, ACC, MIK, YKX, VYY, and MZ provided reagents and mouse models; and LR, RLW, and JLP performed quantitative analyses of the project. All authors read and approved the final manuscript.

## Supplementary Material

Supplemental data

Supplemental video 1

Supplemental video 2

## Version 1 11/22/2022

Electronic publication
