# Supplementary material for: Adenylyl cyclase isoform 1 contributes to sinoatrial node automaticity via functional microdomains
Source: JCI Insight. 2022 Nov 22;7(22):e162602. doi: 10.1172/jci.insight.162602 (PMC9746826; doi:10.1172/jci.insight.162602)
Supplement: Supplemental data [file jciinsight-7-162602-s167.pdf]

Supplementary Materials for

Adenylyl Cyclase Isoform 1 Contributes to Sinoatrial Node Automaticity via Functional Microdomains

Lu Ren[1], Phung N. Thai[1,2], Raghavender Reddy Gopireddy[3], Valeriy Timofeyev[1], Hannah A. Ledford[1], Ryan L. Woltz[1,2], Seojin Park[4], Jose Puglisi[5], Claudia M. Moreno[6,7], Luis Fernando Santana[6], Alana C. Conti[8], Michael I. Kotlikoff[9], Yang Kevin Xiang[2,3], Vladimir Yarov-Yarovoy[6], Manuela Zaccolo[10], Xiao-Dong Zhang[1,2], Ebenezer N. Yamoah[4], Manuel F Navedo[3], Nipavan Chiamvimonvat[1,2]

***Corresponding authors:** Email: nchiamvimonvat@ucdavis.edu, mfnavedo@ucdavis.edu, and enyamoah@gmail.com

This PDF file includes:

Materials and Methods
Figs. S1 to S14
Tables S1 to S2

MATERIALS AND METHODS

Animal Models

Male and female WT, $AC_I^{-/-}$, and $AC_{VIII}^{-/-}$ mice (1, 2) 10-15 weeks old in C57Bl6/J background were used. Mice were housed individually in 12 hr light / 12 hr dark environment. The present investigation conforms to the Guide for the Care and Use of Laboratory Animals published by the US National Institutes of Health (NIH publication No. 85-23, revised 1985) and was performed in accordance with the protocols and guidelines approved by the Animal Care and Use Committee of the University of California, Davis. All experiments were performed in a blinded fashion by different investigators conducting animal handlings, cardiomyocyte isolations, data collection, and analyses.

SAN Cell Isolation

SAN cells were isolated as described (3-7). Mice were anesthetized by intraperitoneal injection of 80 mg/kg of ketamine and 5 mg/kg of xylazine. The heart was excised and placed into Tyrode's solution (35°C) containing (in mM): 140 NaCl, 5.0 HEPES, 5.5 Glucose, 5.4 KCl, 1.8 $CaCl_2$, and 1.0 $MgCl_2$ (pH 7.4). The SAN tissue was dissected based on the landmarks defined by the orifice of superior vena cava, crista terminalis, and atrial septum (8). SAN tissue was digested in low Ca^{2+} solution containing (in mM): 140 NaCl, 5.0 HEPES, 5.5 Glucose, 5.4 KCl, 0.2 $CaCl_2$ and 0.5 $MgCl_2$, 1.2 KH_2PO_4, 50 taurine, pH 6.9, with collagenase B (0.54 U/mL, Sigma-Aldrich, St. Louis, MO), elastase (18.9 U/mL, Sigma-Aldrich) and protease type XIV (1.79 U/mL, Sigma-Aldrich) for 30 mins at 37 °C. After digestion, the tissue was washed three times with Kraft-Bruhe medium containing (in mM): 100 potassium glutamate, 5 HEPES, 20 glucose, 25 KCl, 10 potassium aspartate, 2 $MgSO_4$, 10 KH_2PO_4, 20 taurine, 5 creatine, 0.5 EGTA, and 1 mg mL^{-1} BSA (pH 7.4). SAN cells were dissociated with a transfer pipette by mechanically stirring and pipetting

the tissue chunks. Dissociated SAN cells were used for experiments at room temperature (RT, 22-25°C) or 36 ± 0.5 °C.

Single-cell RT-qPCR

Single cells were identified and isolated with patch pipettes under a microscope. RNA was isolated from single cells using Single Cell-to-CT™ qRT-PCR Kit (ThermoFisher Scientific, Waltham, MA). Single-strand cDNA was synthesized using Superscript III. The quantitative real-time reverse transcription-polymerase chain reaction (qPCR) was performed using predesigned TaqMan Gene Expression assays probes (Thermo Fisher Scientific), *Adcy 1* (Hs00299832_m1), *Adcy 2* (Hs01058848_m1), *Adcy 3* (Hs01086502_m1), *Adcy 4* (Hs00934099_m1), *Adcy 5* (Hs02890018_m1), *Adcy 6* (Hs00209600_m1), *Adcy 7* (Hs00181579_m1), *Adcy 8* (Hs00905042_m1), *Adcy 9* (Hs00181599_m1), and *HCN4* (Hs00975492_m1).

Single-molecule fluorescence in situ hybridization (smFISH)

SmFISH was performed as previously described (9) in WT and AC_I KO SAN sections using probes for AC_I, AC_V, and AC_{VI}. Isolated samples were incubated with 4% diethylpyrocarbonate (DEPC)-paraformaldehyde (PFA) for 24hr, followed by incubation with 30% sucrose for 2-3 days. Samples were then embedded in the optimal cutting temperature (OCT) compound and cryo-sectioned (10 µm) onto superfrost slides. RNAscope multiple fluorescent detection reagents v2 (Advanced Cell Diagnostics, ACD, #323111, Newark, CA) were used for the assay. Probe hybridisation was performed according to the manufacturer's instructions (ACD). Sections were immersed in 4% PFA for 15 minutes at 4°C and serially dehydrated in 50%, 70%, and 100% ethanol for 5 minutes at RT. Sections were then treated with hydrogen peroxidase for 10 minutes at RT, followed by proteinase digestion using protease 4 for 30 minutes at RT. The following steps were performed at 40 °C. Probes were reacted for two hours. AMP1, AMP2,

AMP3 reagents were used to amplify the signal. The appropriate horseradish peroxidase (HRP) reagent and Opal dye were used. HRP blocker was then added to complete the reaction. Slides were washed twice after every step at RT. Probes for *Adcy1* (#451241), *Adcy6* (#539861), *GAPDH* (#4471841), and controls were obtained from ACD. For subsequent immunofluorescent staining, slides were treated with 10% goat serum for 30 mins at RT, incubated with primary antibody specific for HCN4 (1:500 dilution, Alomone Labs, Jerusalem, Israel) overnight at 4°C, washed with TBS-0.005% Tween20 three times for 5 mins each, incubated with secondary antibody (fluorescein isothiocyanate (FITC), ThermoFisher Scientific, 1:500 dilution) for 2 hours at RT, and again washed with TBS-0.005% Tween20 three times for 5 mins each. Slides were incubated in 4′,6-diamidino-2-phenylindole (DAPI) solution for 30 secs at RT to label cell nuclei. They were then mounted on Fluoromount-G and sealed under a coverslip.

Whole-mount Immunohistochemistry

Whole-mount Immunohistochemistry was performed as described previously(10, 11). SAN tissue was dissected and fixed with 4% PFA for 30 min and was then dehydrated through a graded ethanol series (25, 50, 75, 95, 100%), cleared and bleached for 2 hours with 20% dimethyl sulfoxide (DMSO) in ethanol and 12 hours with hydrogen peroxide (6%) in ethanol. Tissue was then rehydrated through a graded ethanol series, washed in PBS (3 × 10 min). SAN tissue was permeabilized with 0.5% Triton X-100 in PBS for 30 mins, blocked for 2 h with 5% normal donkey serum at RT and then incubated with primary antibodies in PBS at 4°C. The following primary antibodies were used: (1) anti-HCN4 (Abcam, Cambridge, MA, 1:200 dilution), a polyclonal antibody raised against rat HCN4, (2) anti-AC_I (Santa Cruz Biotechnology, Inc., Dallas, TX, 1:100 dilution), a monoclonal antibody raised against mouse AC_I. SAN tissue was washed with PBS (3 x 10 minutes) and then incubated with anti-rat and anti-mouse secondary antibodies (Jackson

ImmunoResearch Laboratories, Inc., West Grove, PA, 1:1000 dilution) for 4 hours at RT in the dark. It was then washed in PBS (3 × 10 min) and incubated for 2 h with 20% DMSO diluted in PBS. Coverslips were mounted on the slides with ProLong Diamond Antifade Mountant (ThermoFisher Scientific Inc.). The slides were sequentially imaged using a Zeiss 900 confocal laser-scanning microscope equipped with an Airyscan detector module, a Plan-Apo 63× 1.4 NA oil-immersion objective and 488/561 lasers. Imaris software (Bitplane, Zürich, Switzerland) was used to perform 3D reconstructions.

Echocardiography

Echocardiography to assess systolic and diastolic function were performed using Vevo 2100 (VisualSonics, Fujifilm, Toronto, ON, Canada) imaging system and a MS 550D probe (22–55 MHz) (12, 13). Systolic function was assessed using M-mode and two-dimensional measurements in conscious mice. Diastolic function was assessed in mice anesthetized with 1% isoflurane. The measurements represented the average of six selected cardiac cycles from at least two separate scans performed in a blinded fashion with papillary muscles used as a point of reference for consistency in level of scan. End diastole was defined as the maximal left ventricular (LV) diastolic dimension and end systole was defined as the peak of posterior wall motion. Fractional shortening (FS), a surrogate of systolic function, was calculated from LV dimensions as follows: Fractional shortening (FS), a surrogate of systolic function, was calculated from LV dimensions as follows: FS = ((EDD-ESD)/EDD) x100%, where EDD and ESD are LV end diastolic and end systolic dimension, respectively. A pulsed Doppler velocity profile of inflow across the mitral valve (MV) was performed to assess the diastolic function. E/A ratio (E wave/A wave) was quantified to define diastolic function.

Hemodynamic monitoring

Mice were anesthetized by intraperitoneal injection of 80 mg/kg of ketamine and 5 mg/kg of xylazine and maintained at 37 ºC. Hemodynamic monitoring was performed as previously described (14). The arterial catheter was inserted retrogradely into the left ventricle via carotid artery. The recording of pressure and volume was performed by using Millar Pressure-Volume System MPVS-300 (Millar, Inc., Houston, TX), Power Lab, and Lab Chart 6.0 software (AD Instruments, Colorado Springs, CO). The pressure and volume were calibrated before recordings. The volume calibration used fresh heparinized 37 °C mouse blood and a cuvette (P/N 910-1049, Millar, Inc.). To change the preload, a gentle and quick abdominal compression was applied to occlude inferior vena cava.

Electrocardiography (ECG) Telemetry

All telemetry placements were performed 1 week before the start of each experiment. Mice were anesthetized with ketamine/xylazine (80 mg/kg /5 mg/kg) before placement of a transmitter (Data Sciences International (DSI), New Brighton, MN) into the abdominal cavity with subcutaneous electrodes in the lead I configuration. Baseline measurements were recorded for 24 hours and followed by intraperitoneal injection of isoproterenol (ISO, 0.1 mg/kg, IP) in $AC_{I}^{-/-}$, $AC_{VIII}^{-/-}$ and WT animals. Atropine (2 mg/kg, ip) and propranolol (1 mg/kg, ip) were used to block the heart's autonomic control. The analog telemetric ECG signals were digitised at 1 kHz and recorded using PONEMAH software (DSI). R peaks of the ECG signal were detected, and the mean HR was calculated from the RR interval and averaged for 1 min. For baseline recordings, t=0 corresponds to noon, while t=24 corresponds to midnight. HR variability (HRV) was plotted as RR interval (RR-I) against the next RR interval.

SAN-specific CRISPR/Cas9-mediated gene silencing of AC_I

A transgenic mouse model expressing a fluorescent Ca^{2+} indicator (GCaMP8) under the control of the *Hcn4* promoter was previously generated and used for the study (15). CRISPR/Cas9 system containing 3X sgRNA (GeneCopoeia, Rockville, MD) was used to specifically target the AC_I isoform, followed by *in vivo* delivery using liposome and SAN painting technique (16, 17). A vector containing a scrambled sequence was used as control. Both the targeting and control vectors contained mCherry and were encapsulated in liposomes. The liposomal emulsion was delivered onto the SAN region under direct visualization. ECG and echocardiograms were performed 5-7 days after surgery at baseline and after ISO injection. Light Sheet-Based Fluorescence Microscopy (LSFM) was performed in freshly dissected SAN to confirm that the *in vivo* gene delivery was successful. Green fluorescence protein (GFP) and mCherry signals were simultaneously detected during live SAN imaging.

Light Sheet-Based Fluorescence Microscopy (LSFM)

Freshly dissected tissues were placed in normal Tyrode's solution, immersed in 1.5% agarose in a capillary tube, and mounted inside the Lattice Lightsheet 7 microscope (Carl Zeiss, Jena, Germany). During experiments, tissue was maintained at 37 °C and constantly gassed with 95% O_2/5% CO_2. Baseline measurements were taken before the application of 1 µM of ISO. Imaris software (Bitplane, Zürich, Switzerland) was used to perform 3D reconstructions.

Immunofluorescence Confocal Microscopy

Immunofluorescence labeling was performed as previously described (18). Isolated SAN cells were allowed to adhere to coverslips for 10 minutes before fixing with 4% PFA. Cells were then washed with phosphate-buffered saline (PBS, 3 x 10 minutes). Cells were permeabilized for 10 minutes with 1% Triton X-100% and then blocked with 5% donkey serum for 1h at RT. The

following primary antibodies were used to incubate the cells overnight at 4°C: (1) anti-HCN4 (Abcam, Cambridge, MA, 1:300 dilution), a polyclonal antibody raised against rat HCN4, (2) anti-AC_I (Santa Cruz Biotechnology, Inc., Dallas, TX, 1:100 dilution), a monoclonal antibody raised against mouse AC_I and (3) anti-caveolin-3 (1:300, Thermo Fisher Scientific), a polyclonal antibody raised against rabbit caveolin-3. Cells were washed with PBS (3 x 10 minutes) and then incubated with anti-rat, anti-mouse, or anti-rabbit secondary antibodies (Jackson ImmunoResearch Laboratories, Inc., West Grove, PA, 1:500 dilution) for 1 hour at RT. All the antibodies used were diluted in blocking solution with 5% donkey serum. Cells were then washed with PBS (3 x 10 mins). Coverslips were mounted on the slides with ProLong Diamond Antifade Mountant (Thermo Fisher Scientific Inc.). Control experiments performed by incubation with secondary antibody only did not show positive staining under the same experimental conditions. Identical settings were used for all specimens. Cells were sequentially imaged using a Zeiss 900 confocal laser-scanning microscope equipped with an Airyscan detector module, a Plan-Apo 63× 1.4 NA oil-immersion objective and 488/561/647 lasers. Cells for each group were imaged using the same acquisition parameters. Images were background subtracted, pseudo-colored, and analysed offline using ImageJ. Stimulated emission depletion (STED) microscopy was performed on a Leica STED (TCS SP8 STED 3X) microscope with an HC PL APO 100×/1.4 NA STED objective in STED mode (Leica Microsystems, Wetzlar, Germany). Using Huygens professional software, deconvolution was limited to 15 iterations and a signal-to-noise ratio of 4 with a manual evaluation of background intensity.

Electrophysiology

Whole-cell L-type and T-type Ca^{2+} currents ($I_{Ca,L}$ and $I_{Ca,T}$), HCN currents (I_f) and Na^+ current (I_{Na}) were recorded at 36 ± 0.5 °C using conventional whole-cell patch-clamp techniques

(19). Current-voltage relations were assessed before and after the application of ISO (1 μM). Cell capacitance was calculated as the ratio of total charge (the integrated area under the current transient) to the magnitude of the pulse (20 mV). Currents were normalized to cell capacitance to obtain the current density. The series resistance was compensated electronically. In all experiments, a series resistance compensation of ≥85% was obtained. The currents and membrane potentials were recorded using Axopatch 200A amplifier and Digidata 1440 digitizer (Molecular Devices, LLC., Sunnyvale, CA, USA). The signals were filtered at 2 kHz using a 4-pole Bessel filter and digitised at a sampling frequency of 10 kHz for I_{Ca} and I_{Na} and filtered at 1 kHz and digitised at a sampling frequency of 5 kHz for I_f. All experiments were performed using 3 M KCl agar bridges connecting the ground electrode to the recording chamber. Borosilicate glass electrodes were pulled with a P-97 micropipette puller (Sutter Instruments, Novato, CA). The resistance of the electrodes was ~2-3 MΩ when filled with the pipette solutions. Data acquisition and analysis were carried out using pClamp 10 software (Molecular Devices) and Origin Software (OriginLab, Northampton, MA, USA). No leak compensation was used for the recordings. Recordings were obtained from cells with seal resistance of 1-5 gigaohms. Cells with seal resistance less than 1 gigaohm were rejected.

$I_{Ca,L}$ and $I_{Ca,T}$ were recorded using pipette (intracellular) solution containing (in mM): 16 CsCl, 70 Aspartic acid, 1 $MgCl_2$, 10 EGTA, 10 HEPES, 30 tetraethylammonium chloride (TEA-Cl), 0.1 $CaCl_2$, 5 Mg-ATP; pH was adjusted to 7.2 with CsOH (free Ca^{2+}: 2.617 nM; free Mg^{2+}: 0.9974 mM, based on Maxchelator, Stanford University). The bath (extracellular) solution contained (in mM): 110 NMG, 10 HEPES, 10 glucose, 1.8 $CaCl_2$, 1 $MgCl_2$, 4 4-aminopyridine, 30 TEA-Cl, and 5 CsCl, and pH was adjusted to 7.4 with HCl. To record $I_{Ca,L}$, a family of voltage steps of 400 ms from a holding potential of -55 mV with an interpulse interval of 5 seconds and a

10 mV voltage increment was used. To record T-type I_{Ca} ($I_{Ca, T}$), 10 μM nifedipine was added to the bath solution to block $I_{Ca,L}$. A family of voltage steps of 400 ms from a holding potential of -80 mV with an interpulse interval of 5 seconds with a10 mV voltage increment was used.

For I_f recordings, patch pipettes were filled with intracellular solution containing (in mM): 120 potassium aspartate, 25 KCl, 4.0 $MgCl_2$, 10 EGTA, 4.0 K-ATP, 2.0 Na-GTP, 2.0 phosphocreatine, 5.0 HEPES, 1.0 $CaCl_2$, and pH was adjusted to 7.2 with KOH (free Ca^{2+}: 28 nM; free Mg^{2+}: 0.5083 mM). The extracellular solution contained (in mM): 130 NaCl, 5.0 KCl, 2.0 $MgCl_2$, 1.8 $CaCl_2$, 5.0 HEPES, and 5.0 glucose; pH was adjusted to 7.4 with NaOH. 1 mM $BaCl_2$ was added to block K^+ current. Voltage steps were applied for 2.0 s ranging from -140 mV to -40 mV in 10 mV increments at a holding potential of -35 mV.

I_{Na} recordings were performed as previously described (20, 21). The extracellular solution contained (in mM): 10 NaCl; 120 NMDG-Cl; 2 $CaCl_2$; 1.2 $MgCl_2$; 5 CsCl; 10 HEPES; 10 Glucose (pH was adjusted to 7.4 with CsOH). Nifedipine (10 μM) was added to the solution to block $I_{Ca,L}$ and $NiCl_2$ (40 μM) was added to block $I_{Ca,T}$. The pipette solution contained (mM): 10 KCl, 130 CsCl, 10 NaCl, 10 HEPES (pH was adjusted to 7.2 with CsOH). Voltage steps of 50 ms were applied from a holding potential of -120 mV, ranging from -80 mV to 40 mV in 10 mV increments.

Spontaneous APs and AP firing frequencies in single SAN cells were assessed using the perforated patch-clamp technique at 36 ± 0.5 °C. For AP recordings, amphotericin B (240 μg/ml) was added into the pipette solution. Spontaneous APs were recorded in Tyrode's solution containing (in mM): 140 NaCl, 5.0 HEPES, 5.5 Glucose, 5.4 KCl, 1.8 $CaCl_2$, and 1.0 $MgCl_2$ (pH 7.4). The pipette solution contained (in mM): 130 potassium aspartate, 10 NaCl, 10 HEPES, 0.04 $CaCl_2$, 2.0 Mg-ATP, 7.0 phosphocreatine, 0.1 Na-GTP, with pH adjusted to 7.2 with KOH.

Proximity Ligation Assay (PLA)

Colocalization between AC_I and caveolin-3, AC_I and HCN4, AC_I and $Ca_v1.2$, AC_I and RyR-2, AC_I and β_1-AR, and AC_I and β_2-AR were detected by a Duolink In Situ PLA kit (Sigma-Aldrich) (22). Freshly isolated SAN cells were fixed with 4% PFA and washed with PBS. Cells were then permeabilized with 0.25% Triton X-100 (10 min), blocked in 1% bovine serum albumin (BSA) for 30 mins at RT, and incubated overnight at 4 °C with two primary antibodies 1% BSA + 0.25% Triton X-100 PBS solution. The antibodies used were mouse anti-AC_I (1:100; Santa Cruz Biotechnology), rabbit anti-$Ca_V1.2$ (1:200; ThermoFisher Scientific), rabbit anti-HCN4 (1:200; Alomone Labs), rabbit anti-RyR-2 (1:200; Alomone Labs), rabbit anti- β_1-AR (1:200, Thermo Fisher Scientific), and rabbit anti- β_2-AR (1:200, ThermoFisher Scientific) antibodies. Cells incubated with only one primary antibody were used as negative controls. PLA probes (anti-mouse MINUS and anti-rabbit PLUS) were used as secondary antibodies to bind to primary antibodies. Ligase was added to cells to allow hybridization with the probes, and polymerase was added for a rolling circle amplification reaction. Coverslips were mounted on a microscope slide with Duolink mounting medium. The fluorescence signal was detected using a Zeiss confocal LSM 700 microscope. Images were collected at different optical planes (z-axis step = 0.5 mm). The stack of images for each sample was combined into a single-intensity projection image used to analyze the number of puncta/μm^2 per cell. All the data were analyzed in a blinded fashion with the NIH ImageJ software v1.53c.

Whole-cell Ca^{2+} Transient Measurements

IonOptix contraction system (IonOptix LLC, Westwood, MA) was used to detect spontaneous Ca^{2+} transients from single isolated SAN cells. Freshly isolated SAN cells were loaded with 5 µM Fluo-4 AM (F14201, ThermoFisher Scientific) for 15 minutes at RT. Cells were

then perfused with Tyrode's solution (36 ± 0.5 °C) continuously. Baseline measurements were taken before ISO was applied in both WT and $AC_1^{-/-}$ mice. The maximal Fluo-4 fluorescence (F) was measured at peak amplitude and was normalized to the average of baseline fluorescence (F_0). Background fluorescence was subtracted for each recording.

Local Ca^{2+} Release and Ca^{2+} Transient Detection via Confocal Line Scanning

Local Ca^{2+} release and Ca^{2+} transients were quantified as previously described (23). Freshly isolated SAN cells were loaded with 5 µM Fluo-4 AM for 15 mins at RT. Cells were then perfused with Tyrode's solution (36 ± 0.5 °C) continuously. Line-scan images across the whole cell were obtained to quantify local Ca^{2+} signals with 488 nm excitation and 510 nm emission from the SAN cells. Pixel time was 0.76 µs; line time was 0.91 bms. Pinhole was set at 1.00 AU (Airy unit). Baseline recordings were performed before ISO was applied in both WT and $AC_1^{-/-}$ mice.

Culture of SAN Cells

SAN cells were first isolated as described above and maintained in culture as we have previously described (7) (**Supplementary Fig. 12A-C**). We demonstrate that SAN cells maintained in our culture condition retain their elongated morphology and action potential (AP) waveform for up to 40 hours. The culturing condition does not change β-adrenergic-mediated cAMP signal as determined in freshly dissociated and cultured SAN cells from a cardiac-specific cAMP reporter mouse (7).

Glass coverslips (25 mm, size #0, Karl Hecht, Sondheim, Germany) were coated with 100x diluted laminin (Life Technologies, Grand Island, NY) and incubated for 4 hours at 37°C in 5% CO_2. After 4 hours, coverslips were placed in individual wells in a 24-well plate (Falcon, Tewksbury, MA) and washed 3x with sterile PBS (in mM): 137 NaCl, 2.7 KCl, 10 Na_2HPO_4, 1.8 KH_2PO_4, pH = 7.4). Isolated SAN cells were resuspended in M1018 medium (10.7 g/L)

supplemented with 1x penicillin-streptomycin-glutamate (PSG), 4 mM $NaHCO_3$, 10 mM HEPES, 10% fetal bovine serum (FBS), 6.25 μM blebbistatin and plated on the pre-coated laminin coverslips and incubated for 4 hours at 37°C in 5% CO_2, before the media was replaced with serum-free M1018 (24).

Adenoviral Transfection of cAMP Biosensors in SAN Cells and Confocal Imaging

For adenoviral transfection, the media was replaced with 500 μL of serum-free medium containing adenoviral vectors carrying different versions of the FRET-based cAMP Universal Tag for imaging experiments (CUTie) sensor (25). Accordingly, we employed the cytosolic CUTie, the membrane-targeted AKAP79-CUTie, and sarcoplasmic reticulum-targeted AKAP18δ-CUTie. Cells infected with the desired adenoviral vectors were incubated at 37°C with 5% CO_2 for 36 to 40 hours. Adenoviral vectors were produced using the AdEasy system (Qbiogene Inc., Carlsbad, CA).(26) A Zeiss LSM 700 laser scanning confocal microscope paired with a Zeiss 63x oil immersion lens (numerical aperture = 1.4) was used to collect images at different optical planes (z-axis steps: 0.4 μm) of the YFP fluorescence associated with each FRET construct to confirm expression and expected localization.

Fluorescence Resonance Energy Transfer (FRET) Imaging and Quantification

Glass coverslips with SAN cells were transferred to a glass-bottom culture dish (MatTek, Ashland, MA) containing 3 mL PBS at RT. A Leica DMI3000B inverted fluorescence microscope (Leica Biosystems, Buffalo Grove, IL) equipped with a Hamamatsu Orca-Flash 4.0 digital camera (Bridgewater, NJ) controlled by Metaflor software (Molecular Devices, Sunnyvale, CA) acquired phase contrast, CFP, and YFP images. Phase contrast and CFP480 images were collected with 20x and 40x oil immersion objective lenses, while YFP images were collected using only the 40x oil immersion objective lens. Images for FRET analysis were recorded by exciting the donor

fluorophore at 430-455nm and measuring emission fluorescence with two filters (475DF40 for cyan and 535DF25 for yellow). Images were subjected to background subtraction and acquired every 30 s with exposure time of 200 ms for each channel. The donor/acceptor FRET ratio was calculated and normalized to the ratio value of baseline before ISO. Averages of normalized curves and maximal response to stimulation were graphed based on FRET ratio changes. The binding of cAMP to each FRET biosensor increased the ratio of YFP/CFP and was interpreted as an increase in cAMP levels. Experiments were performed at RT.

Western blot

SAN tissue from WT and $AC_I^{-/-}$ mice were flash frozen in liquid nitrogen for western blotting experiments. The same amount of total protein (5 μg) was loaded in each lane. Membranes were blocked in 3% non-fat dry milk (Bio-Rad) in TBST for 1 hour (room temperature) and then incubated with primary antibodies including anti-HCN4 (1:500 dilution, Alomone Labs), anti-β_1-AR (1:1000 dilution, ThermoFisher Scientific), anti-β2-AR (1:1000, ThermoFisher Scientific), anti-GRK-5 (1:1000 dilution, ThermoFisher Scientific), anti-β-arrestin-2 (1:1000 dilution, ThermoFisher Scientific) and anti-GAPDH (1:5000, Abcam) antibodies, all in 3% non-fat dry milk in TBST overnight at 4°C. On the next day, the membranes were incubated with conjugated secondary antibody (Abcam) for 1 hour at room temperature and the bands were visualized using Fujifilm LAS-3000 Imager.

Chemicals

All chemicals were purchased from Sigma-Aldrich (St. Louis, MO, USA) unless indicated otherwise. Laminin (cat no: 23017015) was obtained from Life Technology, blebbistatin (cat no: 13013) from Cayman Chemical (Ann Arbor, MI), 3-isobutyl-1-methylxanthine (IBMX, cat no: 2845) from Tocris Bioscience (Bristol, UK).

Statistical Analysis

Data were analyzed using GraphPad Prism (San Diego, CA) software and presented as mean ± SEM. Data were assessed for potential outliers using the GraphPad Prism Outlier Test and for normality of distribution. Statistical significance was then determined using appropriate unpaired two-tailed Student's t-test, nonparametric tests, one-way analysis of variance (ANOVA) or two-way ANOVA for multiple comparisons with appropriate post hoc test. Two-way ANOVA was followed by Holm–Sidak multiple comparison test. General linear model was used for two-way repeated measures and mixed-effect model was used when there were missing values. $p<0.05$ was considered statistically significant.

Data availability

All data generated or analyzed in this study are included in the main manuscript and/or supplementary Figures. Raw data of images is available upon request. Source data are provided with this paper.

REFERENCES

1. Wu ZL, et al. Altered behavior and long-term potentiation in type I adenylyl cyclase mutant mice. *Proc Natl Acad Sci U S A.* 1995;92(1):220-4.
2. Bosse KE, et al. Calcium/calmodulin-stimulated adenylyl cyclases 1 and 8 regulate reward-related brain activity and ethanol consumption. *Brain Imaging Behav.* 2019;13(2):396-407.
3. Mangoni ME, and Nargeot J. Properties of the hyperpolarization-activated current (I(f)) in isolated mouse sino-atrial cells. *Cardiovasc Res.* 2001;52(1):51-64.

4. Vinogradova TM, et al. Sinoatrial node pacemaker activity requires Ca(2+)/calmodulin-dependent protein kinase II activation. *Circ Res.* 2000;87(9):760-7.

5. Sharpe EJ, St Clair JR, and Proenza C. Methods for the Isolation, Culture, and Functional Characterization of Sinoatrial Node Myocytes from Adult Mice. *Journal of visualized experiments : JoVE.* 2016(116):54555.

6. Fenske S, et al. Comprehensive multilevel in vivo and in vitro analysis of heart rate fluctuations in mice by ECG telemetry and electrophysiology. *Nature Protocols.* 2016;11(1):61-86.

7. Reddy GR, et al. Deciphering cellular signals in adult mouse sinoatrial node cells. *iScience.* 2022;25(1):103693.

8. Zhang Z, et al. Functional Roles of $Ca_v1.3$ (α_{1D}) calcium channel in sinoatrial nodes: insight gained using gene-targeted null mutant mice. *Circ Res.* 2002;90(9):981-7.

9. Yang L, et al. Single molecule fluorescence in situ hybridisation for quantitating post-transcriptional regulation in Drosophila brains. *Methods.* 2017;126:166-76.

10. Grainger N, Guarina L, Cudmore RH, and Santana LF. The Organization of the Sinoatrial Node Microvasculature Varies Regionally to Match Local Myocyte Excitability. *Function (Oxf).* 2021;2(4):zqab031.

11. Bychkov R, et al. Synchronized Cardiac Impulses Emerge From Heterogeneous Local Calcium Signals Within and Among Cells of Pacemaker Tissue. *JACC Clin Electrophysiol.* 2020;6(8):907-31.

12. Li N, et al. Ablation of a Ca^{2+}-activated K^+ channel (SK2 channel) results in action potential prolongation in atrial myocytes and atrial fibrillation. *J Physiol.* 2009;587(Pt 5):1087-100.

13. Thai PN, et al. Cardiac-specific Conditional Knockout of the 18-kDa Mitochondrial Translocator Protein Protects from Pressure Overload Induced Heart Failure. *Sci Rep.* 2018;8(1):16213.
14. Zhang XD, et al. Prestin amplifies cardiac motor functions. *Cell Rep.* 2021;35(5):109097.
15. Lee FK, et al. Genetically engineered mice for combinatorial cardiovascular optobiology. *Elife.* 2021;10.
16. Gao Z, et al. Genetic inhibition of Na^{+}-Ca^{2+} exchanger current disables fight or flight sinoatrial node activity without affecting resting heart rate. *Circ Res.* 2013;112(2):309-17.
17. Swaminathan PD, et al. Oxidized CaMKII causes cardiac sinus node dysfunction in mice. *J Clin Invest.* 2011;121(8):3277-88.
18. Timofeyev V, et al. Adenylyl cyclase subtype-specific compartmentalization: differential regulation of L-type Ca^{2+} current in ventricular myocytes. *Circ Res.* 2013;112(12):1567-76.
19. Hamill OP, Marty A, Neher E, Sakmann B, and Sigworth FJ. Improved patch-clamp techniques for high-resolution current recording from cells and cell-free membrane patches. *Pflugers Arch.* 1981;391(2):85-100.
20. Lei M, et al. Sinus node dysfunction following targeted disruption of the murine cardiac sodium channel gene Scn5a. *J Physiol.* 2005;567(Pt 2):387-400.
21. Hao X, et al. TGF-beta1-mediated fibrosis and ion channel remodeling are key mechanisms in producing the sinus node dysfunction associated with SCN5A deficiency and aging. *Circ Arrhythm Electrophysiol.* 2011;4(3):397-406.
22. Fredriksson S, et al. Protein detection using proximity-dependent DNA ligation assays. *Nat Biotechnol.* 2002;20(5):473-7.

23. Tagirova Sirenko S, et al. Self-Similar Synchronization of Calcium and Membrane Potential Transitions During Action Potential Cycles Predict Heart Rate Across Species. *JACC Clin Electrophysiol.* 2021;7(11):1331-44.

24. Reddy GR, et al. Illuminating cell signaling with genetically encoded FRET biosensors in adult mouse cardiomyocytes. *J Gen Physiol.* 2018;150(11):1567-82.

25. Surdo NC, et al. FRET biosensor uncovers cAMP nano-domains at β-adrenergic targets that dictate precise tuning of cardiac contractility. *Nat Commun.* 2017;8:15031.

26. Luo J, et al. A protocol for rapid generation of recombinant adenoviruses using the AdEasy system. *Nat Protoc.* 2007;2(5):1236-47.

SUPPLEMENTARY FIGURES

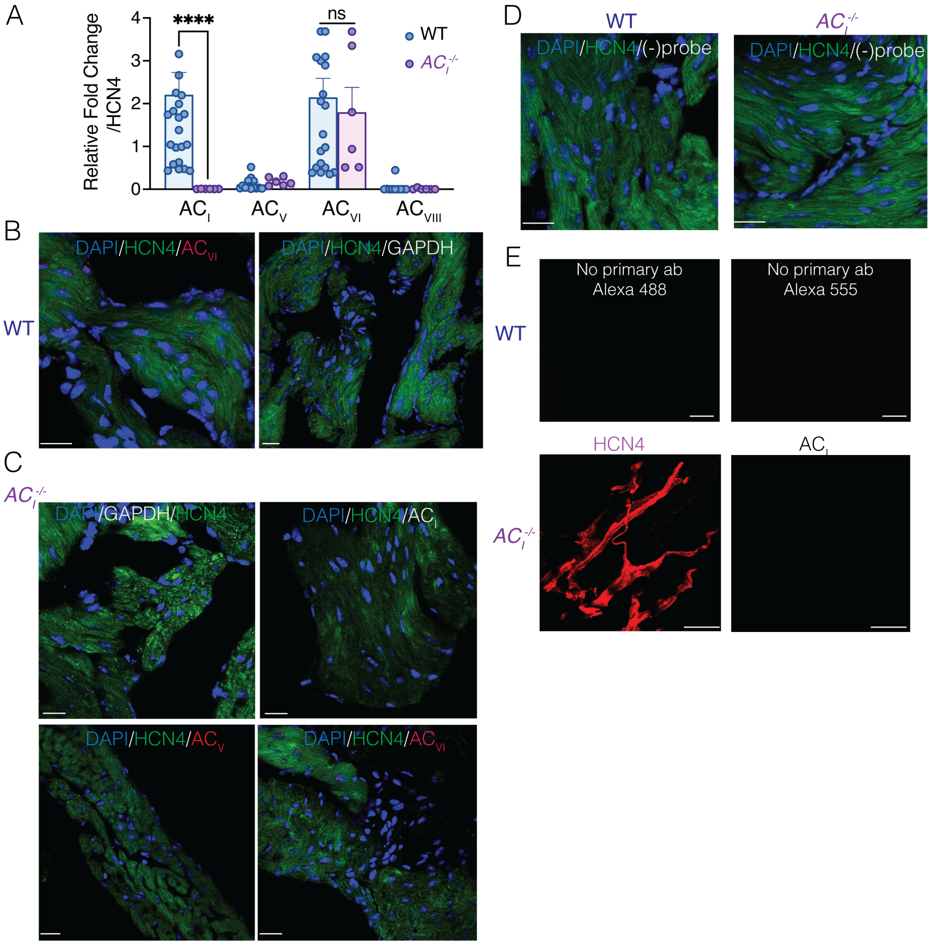

Supplementary Figure S1. **Single-cell RT-qPCR and smFISH of $AC_I^{-/-}$ SANCs**. (**A**) Single-cell RT-qPCR from WT and $AC_I^{-/-}$ SANCs (n=6-23 cells from 2-5 mice). AC_I, AC_V, AC_{VI}, and AC_{VIII} mRNA expressions were normalized to HCN4. Data are expressed as mean ± SEM. ****$p<0.0001$ by unpaired two-tailed t-tests. (**B**) Representative smFISH images of AC_{VI} and GAPDH (positive control) mRNA expressions in the SAN tissue. Between 20-30 cryosections (10 μm) were obtained from SAN tissues. Anti-HCN4 antibodies and DAPI were used to

counterstained the sections. (**C**) Representative smFISH images of GAPDH (positive control), AC_I (negative control), AC_V, AC_{VI} mRNA expressions in the $AC_I^{-/-}$ SAN tissues, showing lack of positive puncta for AC_I mRNA. (**D**) Representative smFISH images of negative control without probes from WT and $AC_I^{-/-}$ SAN tissues. (**E**) Negative control experiments were performed using whole-mount SAN tissues from WT and $AC_I^{-/-}$ mice. Upper panels are whole-mount SAN tissues from WT mice with no primary antibodies (Ab). Lower panels are whole-mount SAN tissues from $AC_I^{-/-}$ mice double stained with anti-HCN4 and anti-AC_I antibodies. Note the lack of signals with anti-AC_I antibody in the $AC_I^{-/-}$ SAN tissue. Scale bar: 20 µm.

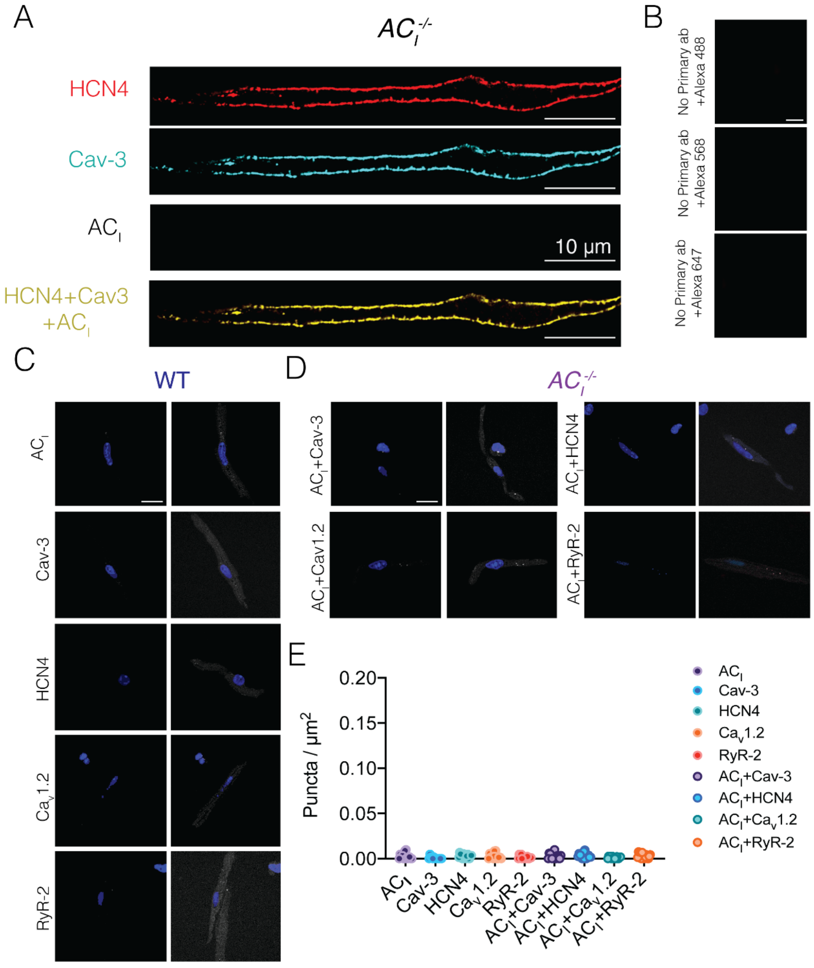

Supplementary Figure S2. **Negative control experiments for immunofluorescence confocal microscopy and proximity ligation assay (PLA)**. (**A**) Representative immunofluorescence confocal microscopic images using $AC_I^{-/-}$ SAN cells as the negative control for anti-AC_I antibody. SAN cells from $AC_I^{-/-}$ were co-stained with anti-HCN4, Cav-3, and AC_I antibodies. The fourth panel represents the merged image. There were no positive signals from anti-AC_I antibody. (**B**) Negative control experiments were also performed using secondary antibodies only in $AC_I^{-/-}$ SAN cells. (**C-D**) Negative control experiments for proximity ligation assays (PLA) where only one antibody was used, showing the lack of positive puncta in WT (**C**) and $AC_I^{-/-}$ (**D**) SAN cells (**E**) Summary data for the negative control experiments for PLA showing the lack of positive puncta. Data expressed as mean ± SEM. Scale bars are 10 μm.

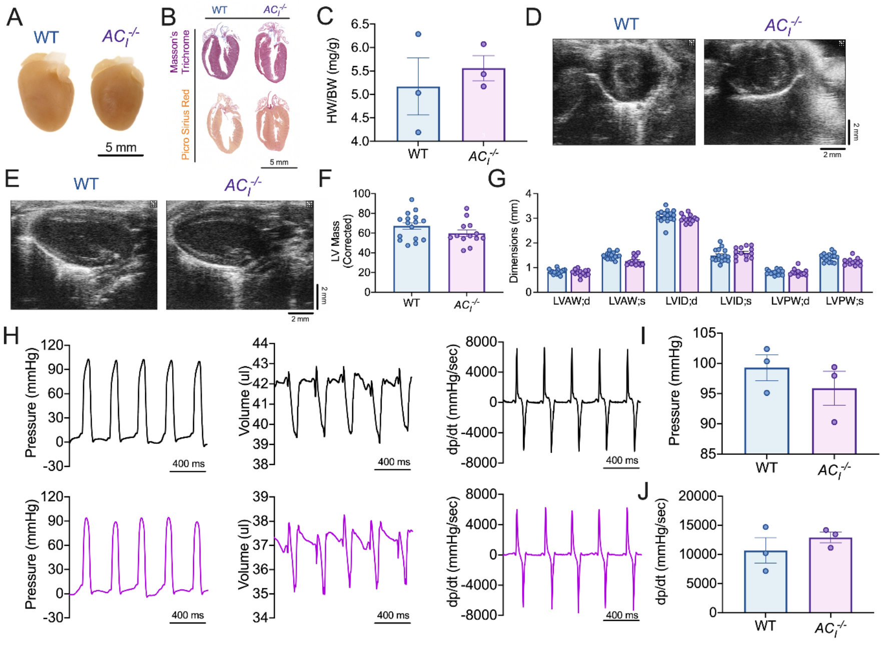

Supplementary Figure S3. **Cardiac structure and function of** $AC_I^{-/-}$ **mice.** (**A**) Representative whole heart images of WT and $AC_I^{-/-}$ mice are shown. (**B**) Hearts were fixed, sectioned, and stained for Masson's Trichrome and Picrosirius red, as depicted. (**C**) Summary data show heart weight (HW) normalized to body weight (BW). Representative echocardiographic images of WT and $AC_I^{-/-}$ mice hearts are displayed at the parasternal short axis (**D**) and (**E**) parasternal long axis. Summary data of corrected left ventricular (LV) mass (**F**) and cardiac dimensions (**G**) are displayed. (**H**) Hemodynamics parameters of pressure, volume, and dp/dt are shown for WT (black) and $AC_I^{-/-}$ mice (purple). (**I**) Summary data of pressure and (**J**) pressure development are shown. Data expressed as mean ± SEM. There were no significant differences between the two groups. Number of symbols in graph represent the number of mice used for the experiments.

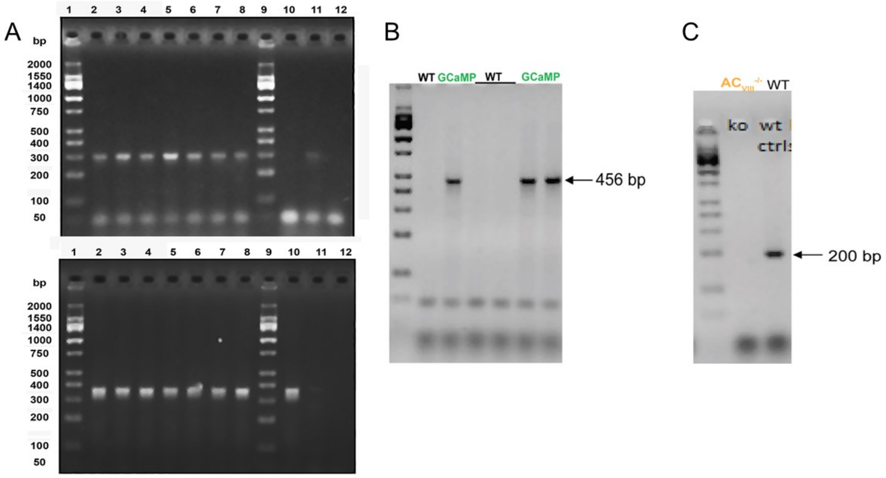

Supplementary Figure S4. **Genotype analyses of WT, $AC_I^{-/-}$, $AC_{VIII}^{-/-}$ and GCaMP8 mice.** (**A**) Photomicrographs of agarose gel from genotype analyses using primers to amplify the WT and mutant bands shown in the upper and lower panels, respectively. Lanes 1 and 9 are All Purpose LO^{TM} DNA Marker 50-2000 bp (Bionexus). Lanes 2-8 are PCR products amplified from genomic DNA samples from heterozygous ACI-/- mice showing WT and mutant bands in the upper and lower panels, respectively. Lanes 10 and 11 are control samples from mutant and WT mice, respectively. Lane 12 is a no template control. The expected sizes for the PCR products are 300 and 370 bp for WT and mutant bands, respectively. (**B**) Photomicrographs of agarose gel from genotype analyses using primers to amplify the transgene from GCaMP8 mice, showing a band at 456 bp for GCaMP8 mice, while WT mice did not exhibit a band. (**C**) Photomicrographs of agarose gel from genotype analyses using primers to amplify the WT band from WT compared to $AC_{VIII}^{-/-}$ mice, showing a band at 200 bp for WT mice, while $AC_{VIII}^{-/-}$ mice did not exhibit a band. Verification of the genotype was always performed prior to the experiments.

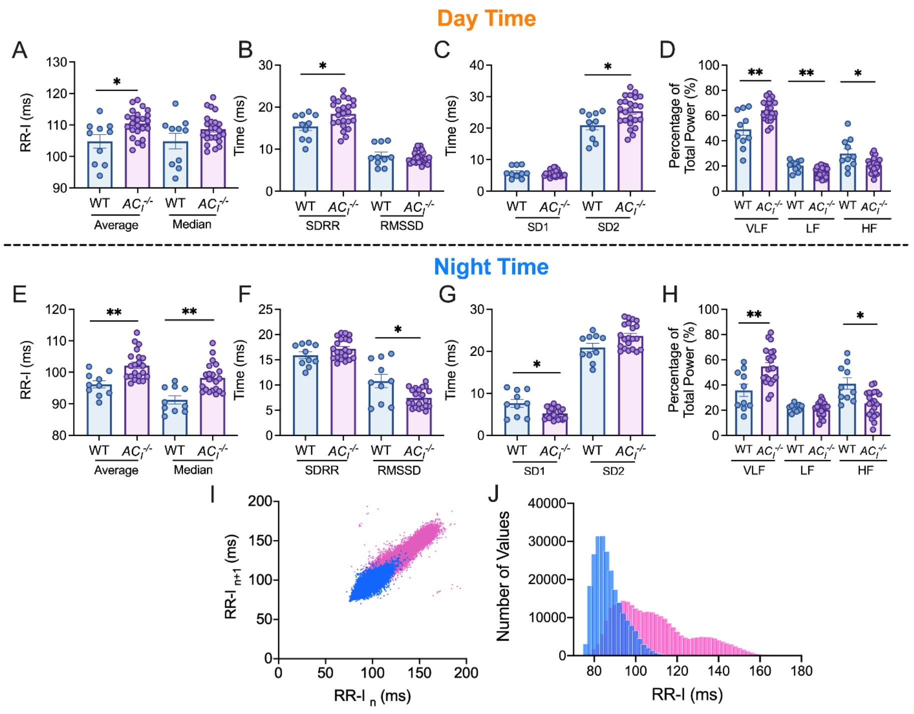

Supplementary Figure S5. Heart rate variability (HRV) of $AC_1^{-/-}$ mice. (**A**) Summary data of the average and median RR-Interval, (**B**) standard deviation of RR-I (SDRR) and root mean square of successive RR interval differences (RMSSD), (**C**) standard deviation (SD) 1 and 2, (**D**) and very-low frequency (VLF), low-frequency (LF), and high-frequency (HF) bands in WT and $AC_1^{-/-}$ mice acquired from day time data (7AM-7PM). Similarly, summary of the (**E-H**) same parameters are shown for data obtained at night (7PM-7AM). (**I**) HRV scatter plot of is shown, as well as the (**J**) histogram showing the distribution of the RR-I. Data expressed mean ± SEM. *$p<0.05$, ** $p<0.01$ by unpaired student's t test, nonparametric test, Mann-Whitney test. Number of points in graph represent the number of mice used for the experiments.

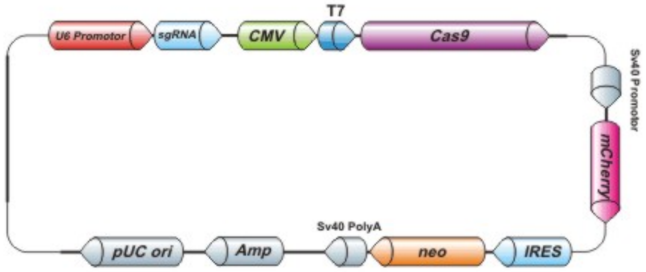

Supplementary Figure S6. A map of all-in-one sgRNA plasmid for mouse *adcy1* gene. mCherry was used as the reporter gene. The plasmid contained ampicillin resistance gene. The targeting sites are: GGGGCGCCGCGCGGCCAAGG; GGAGTTCGCGTGCCCCGAGC; and CCGCGGCTACACGTTGC GGC.

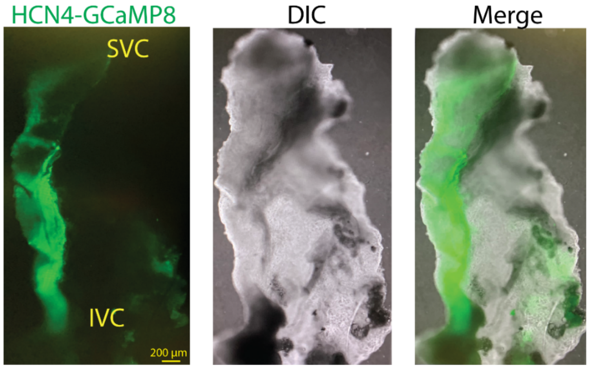

Supplementary Figure S7. Ca^{2+} signal from the SAN region imaged using Lightsheet microscopy from the right atrial tissue from a HCN4-GCaMP8 transgenic mouse. Left panel: Ca^{2+} signal from the SAN region from the HCN4-GCaMP8 mice; middle panel: differential interference contrast image (DIC); right panel: merged image. SVC: superior vena cava; IVC: inferior vena cava.

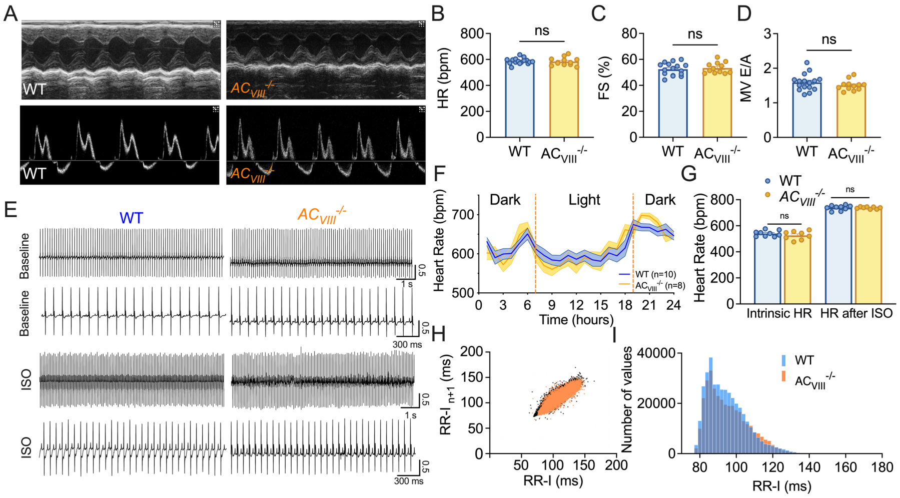

Supplementary Figure S8. Cardiac structure and function of $AC_{VIII}^{-/-}$ compared to the WT mice. (**A**) Representative M-mode echocardiography images of WT and $AC_{VIII}^{-/-}$ mice. Summary data from echocardiography for (**B**) heart rate, (**C**) fractional shortening (FS), and (**D**) mitral valve (MV) E/A ratio. (**E**) Representative ECG tracings of WT and $AC_{VIII}^{-/-}$ mice at baseline and after β-AR stimulation. (**F**) Heart rates (bpm) over 24-hour period recordings are plotted for WT and $AC_{VIII}^{-/-}$ mice. (**G**) Summary data of intrinsic HR after atropine and propanolol injections are shown. (**H**) Representative scatter plots of heart rate variability from WT (black dots) and $AC_{VIII}^{-/-}$ (orange dots). (**I**) Histograms of RR intervals (RR-I) from WT (black bars) and $AC_{VIII}^{-/-}$ (orange bars). The darker bars represent the merged data from the two groups. Data are expressed as mean ± SEM. n = 12-16 mice for each group.

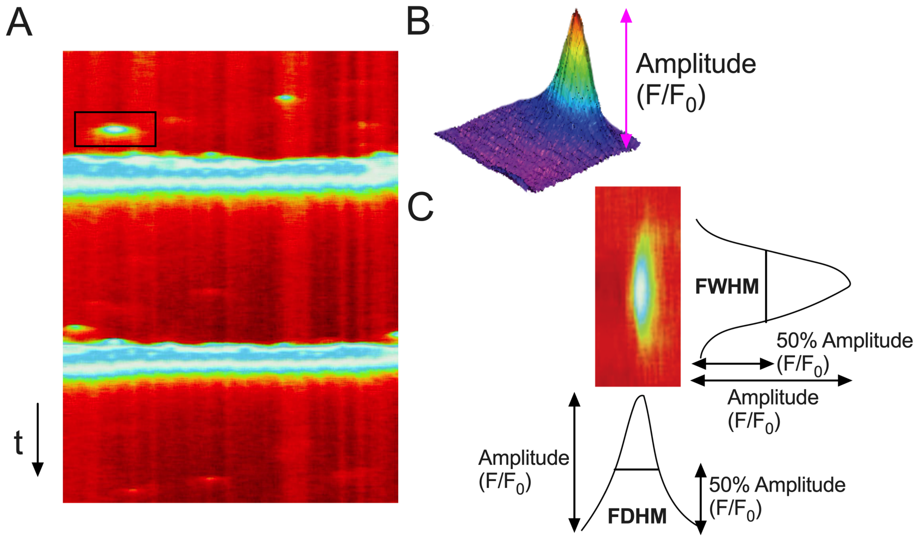

Supplementary Figure S9. (**A**) An example of two-dimensional confocal line-scan of Ca^{2+} transients and LCRs. (**B**) Representative 3D plot of an LCR. (**C**) Surface plots of an LCR, demonstrating the measurements of LCR characteristics (full width at half maximum (FWHM) and full duration at half maximum (FDHM)).

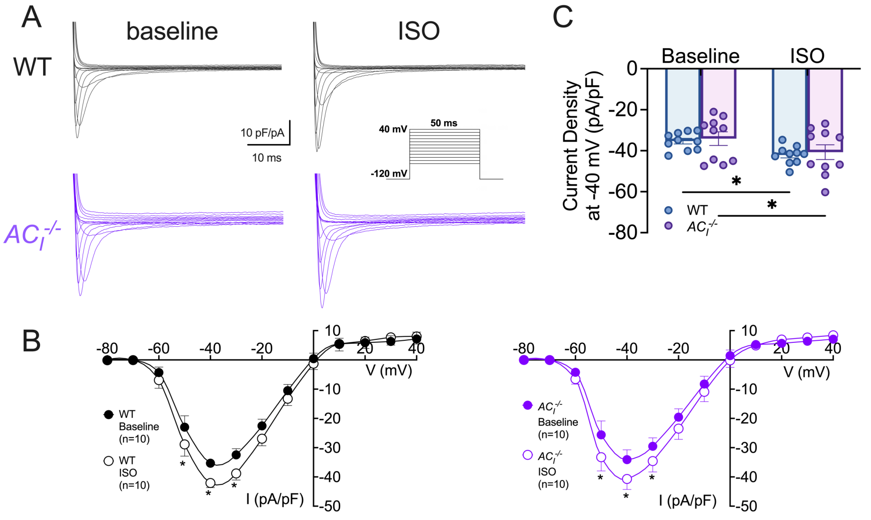

Supplementary Figure S10. Na$^+$ current recordings from WT and $AC_I^{-/-}$ SAN cells. (**A**) Representative traces of I_{Na} for WT and $AC_I^{-/-}$ SAN cells before and after ISO administration. The inset shows a diagram of the voltage-clamp protocol from -80 mV to +40 mV in 10 mV increments from a holding potential of -120 mV. (**B**) Normalized I-V relationship and normalized conductance-voltage relationship of I_{Na} before and after ISO perfusion in WT and $AC_I^{-/-}$ SAN cells. (**C**) Summary data of current density at -40 mV. n=10 cells from 5 mice per group. Data are expressed as mean ± SEM. * p<0.05, by two-way ANOVA, Holm–Sidak multiple comparison post hoc analyses.

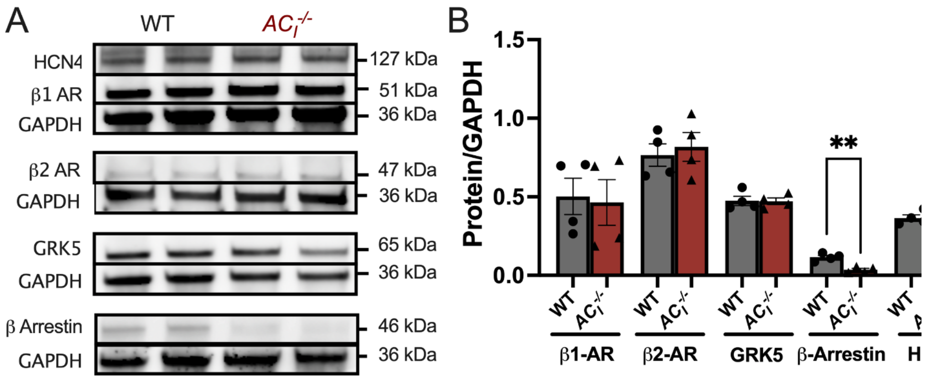

Supplementary Figure S11. Western blot analyses of HCN4, β$_1$-AR, β$_2$-AR, G-protein coupled receptor 5 (GRK5), β-arrestin and GAPDH from WT and $AC_I^{-/-}$ SAN tissues. (**A**) Representative western blot analyses. (**B**) Summary data show the normalized expression of the

proteins (protein/GAPDH). Data expressed mean ± SEM. ** $p<0.01$ by unpaired student's t test, nonparametric test, Mann-Whitney test.

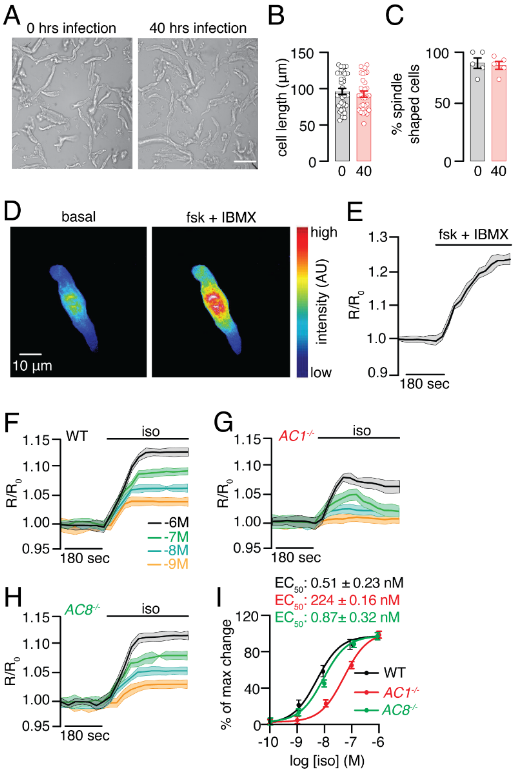

Supplementary Figure S12. cAMP readout using CUTie biosensor in SAN cells. **(A)** Bright field images of adult mouse SAN cells infected with or without cAMP FRET biosensor CUTie at 0 and 40 hours in culture. Scale bar =10 mm **(B)** Histograms of cell length (n = >30 cells from 3 preparations per condition) and **(C)** % of spindle-shaped cells at 0 and 40 hours in culture. (**D**) Representative pseudocolour wide-field FRET ratio images of SAN cells expressing cytosolic CUTie in the absence (left panel) or presence (right panel) of saturating stimulus (forskolin+IBMX). (**E**) Representative time course of changes in the magnitude of normalized FRET responses (R/R_0) in SAN cells expressing cytosol CUTie upon application of forskolin+IBMX. (**F-G**) WT, $AC_I^{-/-}$ and $AC_{VIII}^{-/-}$ SAN cells expressing cytosolic CUTie were stimulated with a set of incremental doses of ISO. Time courses show cytosolic CUTie FRET responses after stimulation with ISO. (**D**) Normalized ISO-induced dose response curves of cytosolic CUTie biosensor (WT, $AC_I^{-/-}$ and $AC_{VIII}^{-/-}$ EC_{50} values are 0.51 ± 0.23 nM, 224 ± 0.16 nM and 0.81 ± 0.32 nM, respectively; *** $p<0.001$ by nonparametric test between WT and $AC_I^{-/-}$). For all experimental sets, data are presented as mean ± SEM. For all experimental sets, n ≥ 8 from at least three biological replicates (independent SAN isolations).

Commented [LR1]: P<0.001 not labeled in the figure since it's for EC50.

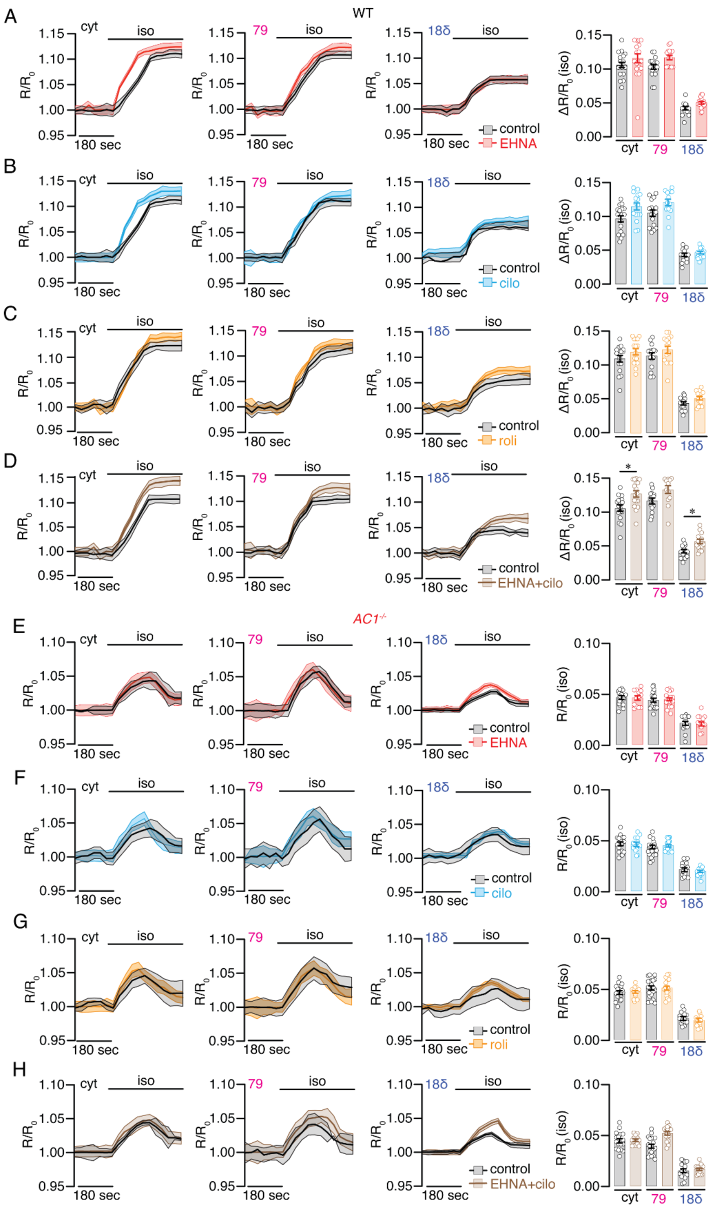
WT
A
B
C
D
E
F
G
H
$AC1^{-/-}$
cyt
79
18δ
iso
180 sec
R/R0
ΔR/R0 (iso)
R/R0 (iso)
control
EHNA
cilo
roli
EHNA+cilo
*
0.95
1.00
1.05
1.10
1.15
0.00
0.05
0.10
0.15

Supplementary Figure S13. Representative time course of changes in the magnitude of normalized FRET responses (R/R_0) in WT SAN cells expressing cytosolic CUTie, AKAP79-CUTie or AKAP18δ-CUTie upon application of ISO (black) in the absence or presence of (**A**) 10 μM EHNA (erythro-9- (2- hydroxy-3-nonyl)adenine, a PDE2 inhibitor), (**B**) 10 μM cilostamide, (**C**) 10 μM rolipram, (**D**) or 10 μM EHNA + cilostamide. Representative time course of changes in the magnitude of normalized FRET responses (R/R_0) in $AC_I^{-/-}$ SANCs expressing cytosolic CUTie, AKAP79-CUTie or AKAP18δ-CUTie upon application of ISO (black) in the absence or presence of (**E**) 10 μM EHNA, (**F**) 10 μM cilostamide, (**G**) 10 μM rolipram, (**H**) 10 μM EHNA + cilostamide. Data expressed as mean ± SEM. *p<0.05 by Kruskal-Wallis with Dunn's multiple comparisons. Statistical differences were compared between all data sets, and the asterisk highlights those with significance. For all experimental sets n ≥ 10 from at least three biological replicates (independent SAN isolations).

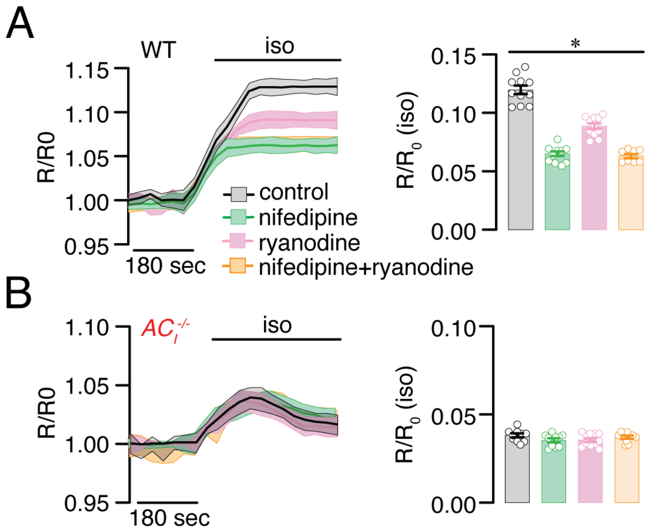

Supplementary Figure S14. Ca^{2+} sources for the activation of ACI isoform: blockade of $I_{Ca,L}$ and SR Ca^{2+} release altered ISO-induced cAMP responses in wild-type but not $AC_I^{-/-}$ SAN cells. Representative time course of changes in the magnitude of normalized FRET responses (R/R_0) in (**A**) WT and (**B**) $AC_I^{-/-}$ SAN cells expressing cytosolic CUTie, upon application of ISO (black) and SAN cells treated with 1µM nifedipine (green), 1µM ryanodine (aqua) or nifedipine+ryanodine (combi; orange) for 20 min. Bar graphs represents the maximal increases in the FRET ratio of these sensors. *p<0.05 by Kruskal-Wallis with Dunn's multiple comparisons. Statistical differences were compared between all data sets, and the asterisk highlights those with significance. For all experimental sets, data are presented as mean ± SEM. For all experimental sets n ≥ 8 from at least three biological replicates (independent SAN isolations).

Supplementary Table S1. Test for normal distribution for Figure 6. Anderson-Darling test was used.

Test for Normal Distribution for Figure 6					
**Panel**		WT baseline	WT ISO	*ACi*$^{-/-}$ baseline	*ACi*$^{-/-}$ ISO
**Panel E**	A2*	0.1796	0.5247	0.2188	0.4884
**Current density at -10 mV**	P value	0.8884	0.1348	0.7762	0.17
	Passed normality test (alpha=0.05)?	Yes	Yes	Yes	Yes
**Panel J**	A2*	0.5366	0.2867	0.4239	0.3238
**Current density at -20 mV**	P value	0.1248	0.5446	0.2536	0.4597
	Passed normality test (alpha=0.05)?	Yes	Yes	Yes	Yes

Supplementary Table S2. Test for normal distribution for Figure 7. Anderson-Darling test was used.

Test for Normal Distribution for Figure 7					
**Panel**		**WT baseline**	**WT ISO**	***$AC_1^{-/-}$* baseline**	***$AC_1^{-/-}$* ISO**
**Panel C**	A2*	0.4523	0.5813	0.9971	0.3241
**Current density**	P value	0.2238	0.0962	0.0087	0.4744
	Passed normality test (alpha=0.05)?	Yes	Yes	Yes	Yes
**Panel E**	A2*	0.2504	0.1463	0.3357	1.156
**$V_{1/2}$**	P value	0.678	0.9454	0.4544	0.003
	Passed normality test (alpha=0.05)?	Yes	Yes	Yes	No
**Panel F**	A2*	0.4962	0.2796	0.3334	0.1942
**Slope factor**	P value	0.1709	0.5539	0.4431	0.8586
	Passed normality test (alpha=0.05)?	Yes	Yes	Yes	Yes
**Panel G**	A2*	0.3431	0.5712	0.301	0.2956
**Tau fast**	P value	0.4258	0.1025	0.528	0.5169
	Passed normality test (alpha=0.05)?	Yes	Yes	Yes	Yes
**Panel H**	A2*	0.3838	0.2825	0.6698	0.3671
**Tau slow**	P value	0.3416	0.5752	0.0596	0.3473
	Passed normality test (alpha=0.05)?	Yes	Yes	Yes	Yes
	A2*	0.4285	1.124	0.3135	1.014
